# Lattice Strain and Built‐In Field Synergistically Boost Urea Oxidation via Dynamic NiOOH Mediation at Multiphase Heterointerfaces

**DOI:** 10.1002/adma.74790

**Published:** 2026-09-01

**Authors:** Jiawei Li, Liancen Li, Guangfu Qian, Zishan Cai, Yiping Cao, Yihao Xu, Ruyu Zhang, Jinli Chen, Changzhou Chen, Panagiotis Tsiakaras

**Affiliations:** ^1^ Guangxi Key Laboratory of Clean Pulp & Papermaking and Pollution Control School of Light Industry and Food Engineering Guangxi University Nanning China; ^2^ State Key Laboratory of Materials Processing & Die and Mould Technology School of Materials Science and Engineering Huazhong University of Science and Technology Wuhan China; ^3^ Laboratory of Alternative Energy Conversion Systems Department of Mechanical Engineering School of Engineering University of Thessaly Pedion Areos Volos Greece

**Keywords:** built‐in electric field, lattice strains, multiphase heterogeneous interfaces, sulfur‐doped lignin‐derived carbon, urea electrolysis

## Abstract

The practical application of nickel‐based heterojunction electrocatalysts for the urea oxidation reaction (UOR) is impeded by insufficient active sites and sluggish multi‐step reaction kinetics. Herein, a sulfur‐doped lignin‐derived carbon encapsulated Ni‐NiO‐MoO_2_ micro‐nano array (Ni‐NiO‐MoO_2_@SC) is fabricated to tackle these issues. Experimental and theoretical results demonstrate that the multiphase heterointerfaces spontaneously generate a built‐in electric field due to the distinct work function and electronic structure of each component, and simultaneously induce lattice strain, which synergistically optimizes the electronic structure and accelerates the dynamic formation of active NiOOH species. In‐situ electrochemical characterizations and density functional theory calculations further confirm that the engineered interface promotes urea adsorption, facilitates N─N coupling and C─N bond cleavage, expedites *COOH intermediate desorption, and avoids excessive accumulation of NiOOH. Consequently, Ni‐NiO‐MoO_2_@SC delivers superior UOR activity with low potentials of 1.34/1.40 V at 10/500 mA cm^−2^, respectively. Moreover, the sulfur‐doped carbon encapsulation and micro‐nano array architecture endow the catalyst with excellent corrosion resistance and mass transport capability, realizing long‐term stability (500 mA cm^−2^ at 1.53 V for 500 h) in membrane electrode assemblies. This work provides a versatile interface engineering and encapsulation strategy for high‐performance nickel‐based electrocatalysts toward urea electrolysis and beyond.

## Graphical abstract

A sulfur‐doped lignin‐derived carbon‐coated Ni‐NiO‐MoO_2_ nanorod array was synthesized, exhibiting enhanced UOR performance ‐ due to lattice‐strain and built‐in electric field effects by multiphase heterogeneous interface in Ni‐NiO‐MoO_2_@SC, excellent corrosion resistance, mass‐transport ability, and long‐term stability (500 mA cm^−2^ at 1.53 V for 500 h) in membrane electrode assemblies.

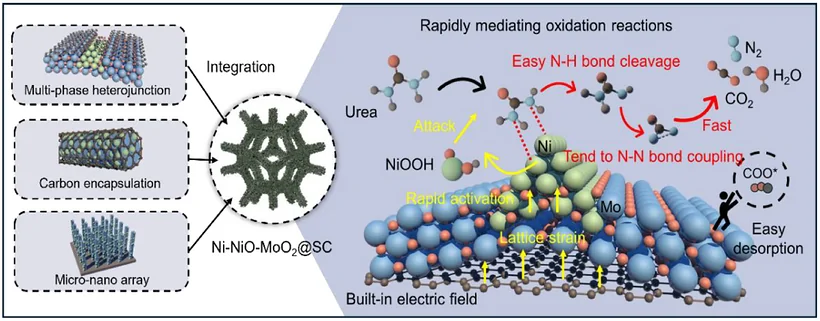

## 1 Introduction

Urea electrolysis, which contains the urea oxidation reaction (UOR) and the hydrogen evolution reaction (HER), is an efficient, clean, and convenient approach to degrading urea‐rich wastewater at the anode while generating hydrogen at the cathode [1], which has been widely recognized by researchers. However, the UOR has sluggish six‐electron reaction kinetics, including processes such as proton‐coupled electron transfer (PCET), N─N bond coupling, C─N bond cleavage, and adsorption/desorption of intermediates, which result in low reaction activity and elevated reaction potentials [2, 3]. Additionally, the chemical and mechanical stability of the systems needs further improvement under both low and high current densities due to electrolyte‐induced electrode corrosion and the accumulation of CO_2_ and N_2_ gases on electrode surfaces [4, 5]. Therefore, developing high‐performance catalysts to accelerate the UOR process is crucial.

Nickel‐based catalysts with properties similar to those of scarce noble metals (Pt/Ir/Ru) are ideal materials for UOR. However, the traditional nickel‐based catalysts are prone to excessive accumulation of NiOOH species during the reaction process, resulting in passivation of the active site and blockage of the reaction kinetics. Meanwhile, under high current conditions, the catalyst is prone to electrolyte corrosion and reaction intermediate poisoning; the structure is easy to collapse, and performance decays rapidly [6, 7]. To address this, biphasic nickel‐based heterojunction catalysts have been widely studied, which can optimize the UOR pathway through charge redistribution to alleviate the above defects to a certain extent [8, 9, 10]. Yet, their unidirectional electron transfer restricts active site density and availability [11, 12], which cannot coordinate the six‐electron reaction, causing sluggish UOR kinetics (Figure 1a).

**FIGURE 1:**
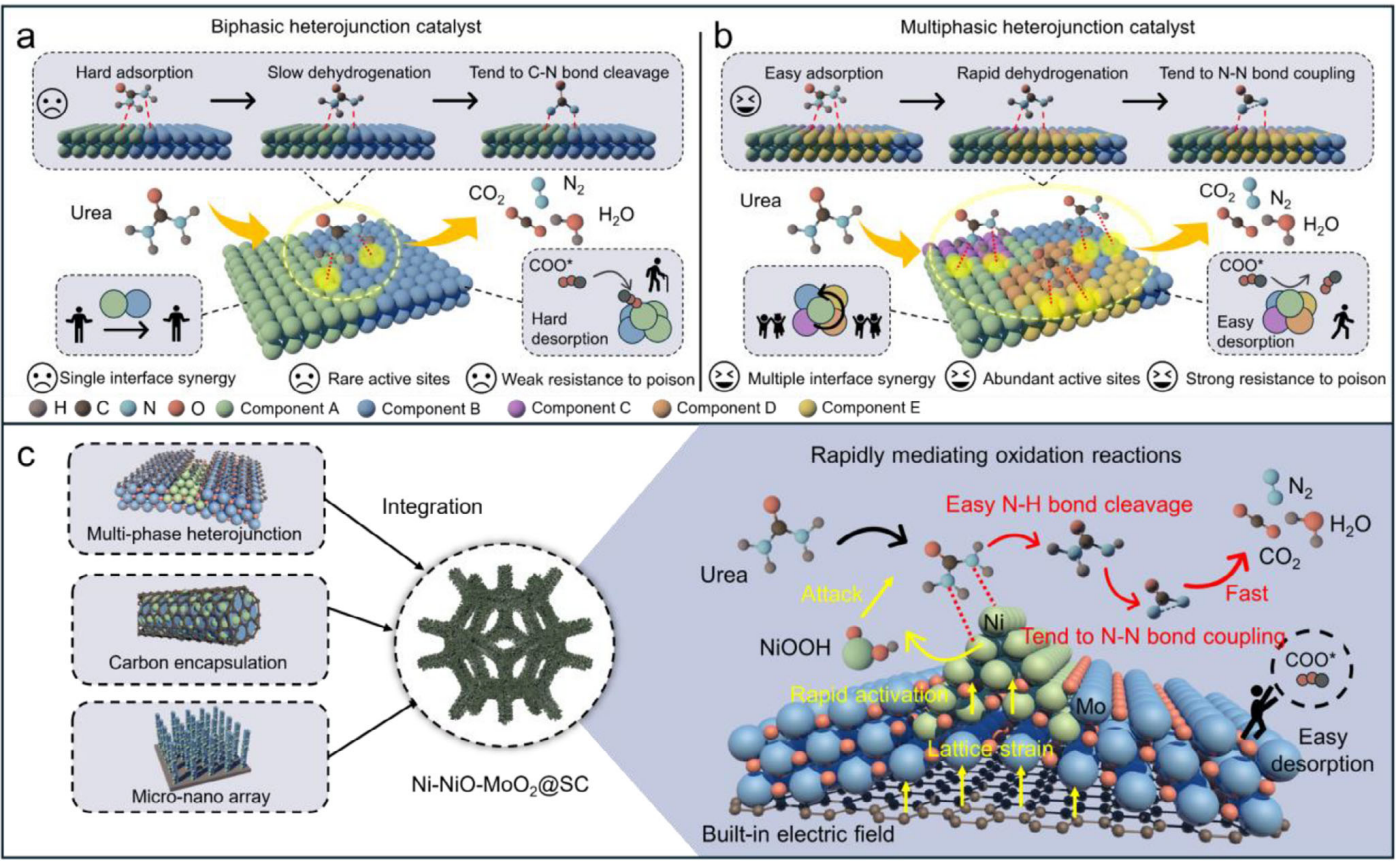
(a–c) Comparison of biphasic, multiphasic heterojunctions, and Ni‐NiO‐MoO_2_@SC.

In contrast, multiphasic nickel‐based heterojunctions leverage multi‐interface synergy to activate more built‐in electric fields for optimized electronic structures and increase the density of active sites to regulate six‐electron steps, which significantly enhance UOR activity (Figure 1b) [13, 14, 15, 16, 17, 18]. Huang et al. combined experimentation and density functional theory (DFT) calculation demonstrate that Ni(OH)_2_ was the active center in the CuO@Ni(OH)_2_@FeOOH NWs/CF, and the synergistic interaction between multiple components led to the reorganization of the charge of Ni site, thus adjusting its electronic structure and d band center, and thus showing excellent UOR activity (1.37 V at 100 mA cm^−2^, 41.4 mV dec^−1^) [19]. However, three major challenges hinder progress: (1) obscured active phases from surface reconstruction/corrosion, (2) unclear reaction mechanisms due to complex electronic structures, and (3) neglected interfacial strain caused by element interdiffusion. These limitations, prevalent even in advanced nickel‐based heterojunction catalysts, thus underscore the urgent need to concurrently boost both activity and durability in harsh conditions.

Lignosulfonates, with their inherent oxygen and sulfur functional groups, can uniformly coordinate with metals and be converted into sulfur‐doped biochar with rich defect structures upon annealing [20, 21]. Some studies have shown that forming a carbon‐encapsulation structure by combining lignin‐derived carbon with metallic species suppresses metal leaching, optimizes electronic configurations, and shows exceptional corrosion resistance in harsh alkaline conditions, thereby enhancing the catalytic viability of metal‐based catalysts, such as water electrolysis and 5‐hydroxymethylfurfural oxidation [22, 23, 24]. Thus, these results confirm that lignin‐derived carbon can be employed to improve UOR's performance through interfacial synergy and carbon‐encapsulation structure, which imparts uniform active sites, suppressed metal leaching, high conductivity, and poisoning resistance. However, most lignin‐derived carbon catalysts with powdered forms require binders that compromise catalyst‐substrate contact and block mass transport, degrading performance. Our prior work demonstrated that integrating transition metal‐lignin heterostructures into self‐supporting electrodes offers an intrinsic advantage over powdered lignin‐derived carbons by substantially boosting UOR performance [25, 26, 27].

Here, by integrating interface engineering, sulfur‐doped lignin‐derived carbon, and micro‐nano array architecture, the Ni‐NiO‐MoO_2_@SC electrocatalyst was fabricated with multiphase heterogeneous interfaces (Figure 1c). Combined physical characterization and density functional theory (DFT) calculations collectively demonstrate that the multiphase heterojunctions (formed among Ni, NiO, MoO_2_, and sulfur‐doped lignin‐derived carbon) induce lattice strain and built‐in electric fields. These synergistic effects collectively optimize the electronic structure and enhance the adsorption/desorption energetics of reaction intermediates, thereby promoting the rapid dynamics of NiOOH, as confirmed by in situ electrochemical analysis. Furthermore, DFT calculations reveal that the NiOOH‐Ni‐NiO‐MoO_2_@SC configuration preferentially facilitates proton‐coupled electron transfer, N─N bond coupling, C─N bond cleavage, and *COOH desorption, significantly boosting the intrinsic activity of the UOR. Thereby, it demonstrates good UOR activity with low potentials of 1.34/1.40/1.44 V@10/500/1000 mA cm^−2^, 21.45 mV dec^−1^, TOF (16.5 s^−1^ at 1.40 V), and MA (0.91 mA mg^−1^ at 1.40 V). Meanwhile, the sulfur‐doped lignin‐derived carbon encapsulation strategy, synergized with the micro‐nano array architecture to enhance stability, enables Ni‐NiO‐MoO_2_@SC to operate stably as a dual‐function electrode at a current density of 500 mA cm^−2^ for 500 h.

## 2 Results and Discussion

### 2.1 Physical Characterization of Catalysts

The catalyst synthesis (Figure 2a) adopts a two‐step protocol (solvothermal growth followed by thermal annealing). Scanning electron microscopy (SEM, Figures S1 and S2) shows that solvothermal treatment yields uniform rod‐like microarray precursors with smooth surfaces on nickel foam (NF). Subsequent annealing induces structural reconstruction, forming nanoparticle‐decorated microarrays (micro‐nano‐arrays) to produce Ni‐NiO‐MoO_2_@SC (Figure 2b). This configuration synergizes microarray structural stability and nanoparticle high reactivity, enhancing catalytic efficiency via increased active site‐exposing surface area and optimized gas‐liquid transport [28]. Other catalysts (Ni‐NiO‐MoO_2_, MoO_2_@SC, etc.) have distinct morphologies (Figures S3–S7), indicating that sodium lignin sulfonate, ammonium molybdate tetrahydrate, nickel nitrate hexahydrate, and the preparation method critically affect final morphology, which modulates surface area, pore architecture, and hydrophilicity to influence urea electrolysis performance.

**FIGURE 2:**
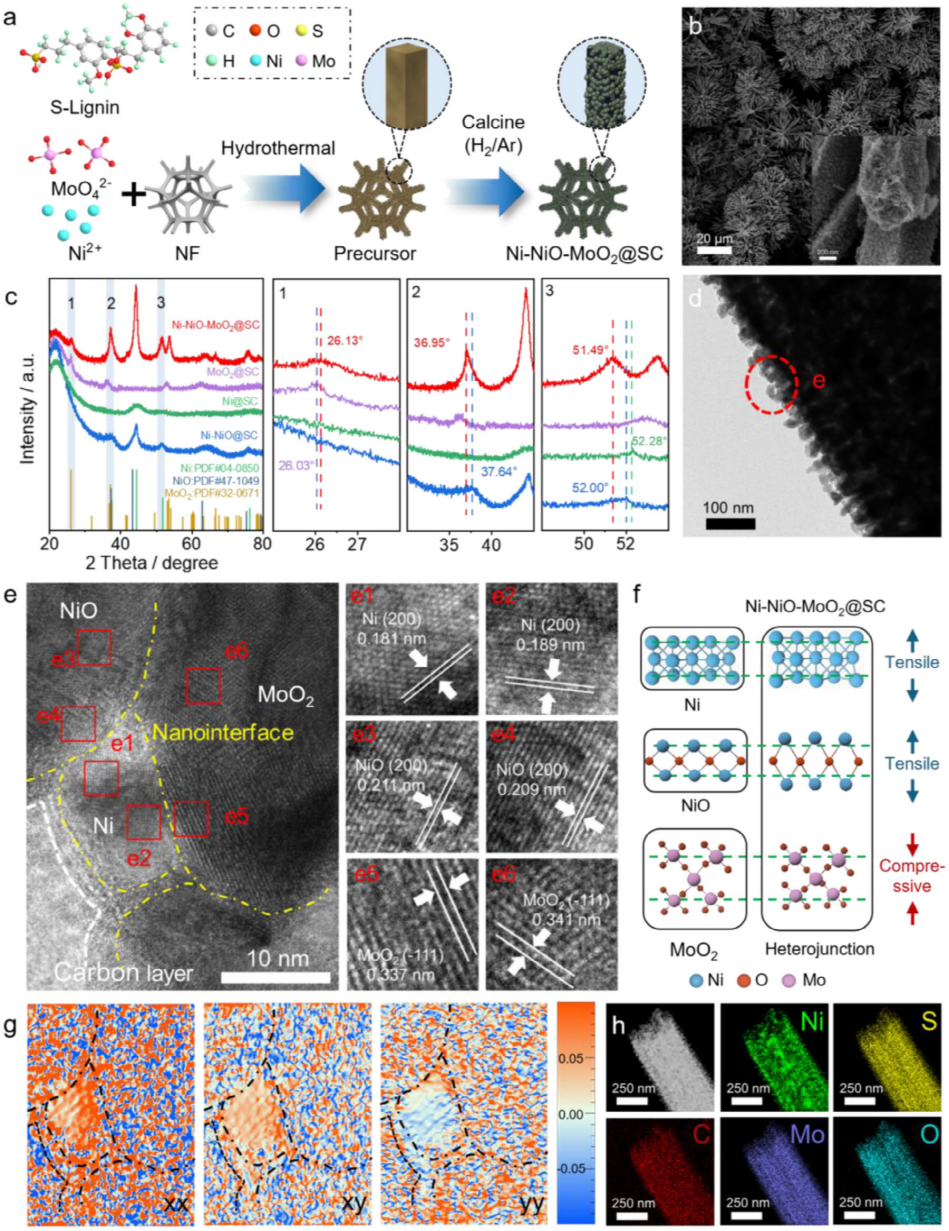
(a) Schematic illustration of the Ni‐NiO‐MoO_2_@SC synthesis; (b) SEM; (c) XRD; (d) TEM; (e) HRTEM; (f) Schematic illustration of lattice evolution in Ni‐NiO‐MoO_2_@SC heterostructure; (g) GPA strain mapping of (e); (h) EDX of Ni‐NiO‐MoO_2_@SC.

Subsequently, Brunauer‐Emmett‐Teller (BET) surface area, pore size distribution, hydrophilicity, and bubble evolution were studied via N_2_ adsorption‐desorption isotherms, contact angle measurements, and in situ optical imaging. In Figures S8–S10, Ni‐NiO‐MoO_2_@SC has a type IV isotherm (surface area: 62.4 m^2^ g^−1^, pore size: 0–41.3 nm), differing from the precursor's type III isotherm. Ni‐NiO‐MoO_2_@SC also has a higher BET surface area than other catalysts, implying lignin‐derived carbon‐integrated micro‐nano‐arrays expand accessible surface area for more active sites. Importantly, dynamic contact angle measurements (Figure S11) show that Ni‐NiO‐MoO_2_@SC enables rapid dissipation of 1.0 KOH + 0.5 urea electrolyte droplets (wetting in 100 ms), exhibiting superior interfacial hydrophilicity and enhanced mass transfer. In situ optical monitoring (Figures S12 and S13, Videos S1 and S2) reveals that Ni‐NiO‐MoO_2_@SC exhibits optimized bubble detachment, while other samples show prolonged coalesced microbubble retention, forming insulating gas blankets that impair electrode‐electrolyte contact.

Crystalline phase analysis via X‐ray diffraction (XRD, Figure 2c) identifies characteristic diffraction signatures of Ni, NiO, and MoO_2_ in both Ni‐NiO‐MoO_2_@SC and its counterpart (Ni‐NiO‐MoO_2_) without sulfur‐doped lignin‐derived carbon (SC), confirming equivalent inorganic compositions. The absence of carbon‐related diffraction peaks suggests amorphous or weakly crystalline carbon formation during pyrolysis. Raman spectroscopy displays distinct D/G‐band vibrational signatures (1300/1580 cm^−1^) in Ni‐NiO‐MoO_2_@SC but is absent in Ni‐NiO‐MoO_2_ (Figure S14). Meanwhile, transmission electron microscopy (TEM) mappings and high‐resolution TEM (HRTEM) further display the existence of the carbon matrix's spatial distribution, verifying the SC incorporation. Additionally, the HRTEM images of another area (Figure S18) indicated that the SC was semi‐encapsulated on the surface of Ni‐NiO‐MoO_2_ in an amorphous form rather than a fully compact or closed core‐shell structure.

It is noted that XRD patterns of Ni‐NiO‐MoO_2_@SC exhibit noteworthy variations in peak positions compared to their binary counterparts (Figure 2c). Specifically, the characteristic peaks corresponding to MoO_2_ in this ternary composite undergo a positive angular shift of 0.1° when contrasted with MoO_2_@SC. A more pronounced negative angular shift of 0.51° for Ni and 0.69° for NiO is observed compared to the corresponding peaks in Ni‐NiO@SC. This systematic displacement, when analyzed through the Bragg equation (Tables S1–S3) [29], indicates the development of compressive‐tensile lattice strains within the Ni, NiO, and MoO_2_ crystallographic planes upon their integration into the hybrid structure. Such strain generation likely originates from differential atomic diffusion processes during material synthesis, attributable to the inherent size disparity between nickel and molybdenum atoms. Prior studies [30] have established that these compressive‐tensile strain configurations can induce controlled lattice distortions, thus enhancing defect densities and creating more active sites beneficial for UOR catalytic activities.

Then, TEM analysis confirms the nanoparticle‐decorated microarray architecture (Figure 2d), aligned with SEM observations, enabling efficient gas‐liquid transfer during urea electrolysis under high‐current‐density conditions. HRTEM (Figure 2e) clearly shows the presence of an amorphous carbon layer, which is favorable for electron transfer and structural stability. Meanwhile, the intimate nanointerfaces among Ni (200), NiO (200), and MoO_2_ (111) induce obvious lattice distortion and electron density redistribution, which could be believed to provide favorable conditions for enhancing the UOR catalytic performance [31]. Significantly, lattice distortion with varying degrees of tensile and compressive strain is observed across the interface regions (Figures 2e1–e6). The interplanar spacing measured for different crystallographic planes demonstrate this structural modulation: Ni (200) exhibits 0.181 and 0.189 nm, NiO (200) displays 0.211 and 0.209 nm, while MoO_2_ (−111) shows 0.337 and 0.341 nm in their respective interfacial zones. That is, the Ni and NiO undergo tensile strain, while the MoO_2_ undergoes compressive strain at the interface (Figure 2f).

Then, geometric phase analysis (GPA, Figure 2g) was employed to quantify interfacial strain effects. Strain tensor mappings (Ɛ_xx_, Ɛ_xy_, and Ɛ_yy_) demonstrate a tensile‐to‐compressive contrast between Ni/NiO regions versus compressive strain in MoO_2_ zones. This strain dichotomy arises from an atomic radius mismatch between Ni (0.124 nm) and Mo (0.136 nm), driving interfacial element interdiffusion that creates alternating tensile‐compressive fields. Such lattice distortions enhance catalytic activity by generating structural imperfections that increase active site density [32]. Elemental mappings (Figure 2h) confirm the homogeneous distribution of C, Ni, S, O, and Mo, with quantitative inductively coupled plasma mass spectrometry (ICP‐MS) analysis (Table S4) validating the elemental content.

Furthermore, to gain deeper insights into the dynamic variations in local coordination and electronic structure of Ni‐NiO‐MoO_2_@SC, X‐ray absorption fine structure (XAFS) measurements were conducted. Figures 3a,b displays the normalized X‐ray absorption near‐edge structure (XANES) spectra at the Ni K‐edge and Mo K‐edge of Ni‐NiO‐MoO_2_@SC, along with those of Ni‐NiO@SC, Ni@SC, MoO_2_@SC, and reference samples for comparative analysis. Compared to Ni‐NiO@SC and Ni@SC, Ni‐NiO‐MoO_2_@SC shows a reduced intensity of the pre‐edge peak at the Ni K‐edge (corresponding to the 1s→3d transition). This phenomenon indicates that interfacial coupling between Ni‐NiO‐MoO_2_ enhances 3d orbital hybridization, optimizes the internal electronic structure, and promotes rapid electron transfer. Moreover, the increased white‐line intensity at the Ni K‐edge indicates that Ni metal states in the Ni‐NiO‐MoO_2_@SC and contrast catalysts are similar but have a more abundant positive charge. In contrast, the white‐line intensity of the Mo K‐edge of Ni‐NiO‐MoO_2_@SC is reduced, which implies a partial accumulation of electrons on the Mo species after the introduction of Ni and NiO components.

**FIGURE 3:**
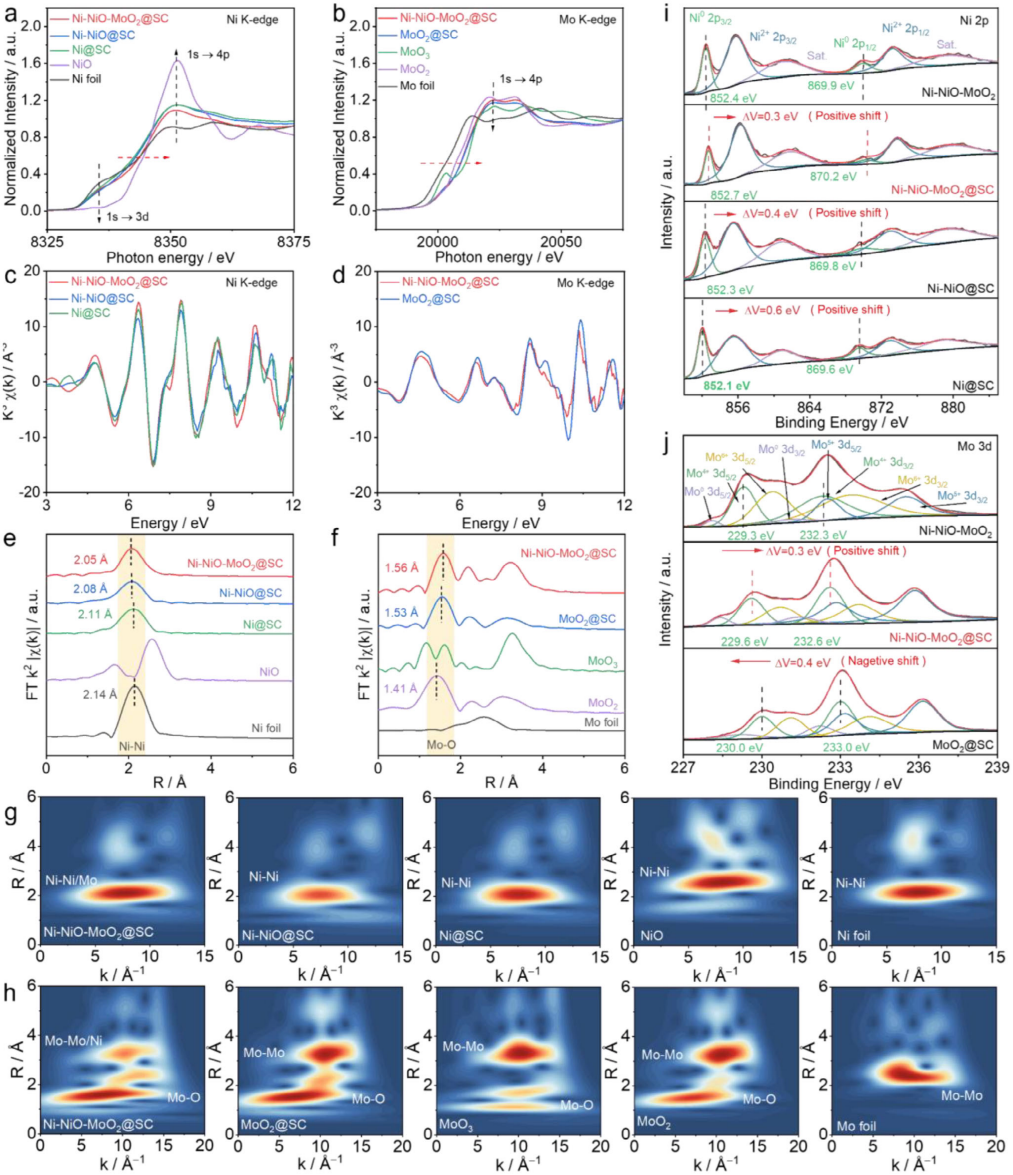
(a‐b) The XANES spectrum in Ni K‐edge and Mo K‐edge, (c‐d) K‐space; (e‐f) R‐space; (i‐j) HRXPS spectra of Ni 2p and Mo 3d; (g, h) the wavelet transforms (WT) contour plots.

A comparative analysis of the X‐ray absorption fine structure (EXAFS) oscillations in k‐space at both the Ni and Mo K‐edges reveals a distinct asymmetric behavior in the Ni‐NiO‐MoO_2_@SC heterostructure (Figures 3c,d): the Ni edge exhibits a slightly enhanced oscillation amplitude relative to the Ni‐NiO@SC reference, whereas the Mo edge displays a noticeable attenuation. This contrasting trend can be coherently explained by asymmetric structural evolution at the heterointerface [33]. The amplitude reduction at the Mo edge signifies increased static disorder and bond length dispersion in the MoO_2_ phase, reflecting substantial local lattice strain as it accommodates coordination with the Ni/NiO lattice. Concurrently, the slight amplification of oscillations at the Ni edge suggests improved local structural order around Ni sites, likely induced by the formation of well‐defined interfacial bonds (e.g., Ni─O─Mo), which effectively suppresses surface disorder of Ni/NiO domains. Together, these results demonstrate that the components of MoO_2_ and Ni/NiO undergo adaptive lattice reconstruction at the heterogeneous interface, jointly inducing a prominent lattice strain effect. This further optimizes the electronic coordination environment and interfacial charge behavior, ultimately improving the intrinsic electronic properties and catalytic reaction activity of the catalyst [34].

To further verify the evolution of the metal coordination environment, the extended EXAFS data were subjected to Fourier transformation, yielding structural information in real space (R‐space, Figures 3e,f). Compared with Ni‐NiO@SC and Ni@SC, Ni‐NiO‐MoO_2_@SC exhibits a shorter apparent Ni─Ni bond length, while the apparent Mo─O bond length is slightly elongated. This indicates a strong interfacial interaction between Ni, NiO, and MoO_2_, which involves electronic coupling and structural rearrangement of the heterojunction, thereby promoting charge redistribution and local lattice modulation. These findings are consistent with the results obtained from TEM observations.

Simultaneously, the Ni K‐edge (Figure 3g), the dominant signal at k ≈ 7.5 Å^−1^, R ≈ 1.98 Å corresponds to the Ni─O first coordination shell, and the peak at k ≈ 10.5 Å^−1^, R ≈ 2.55 Å is assigned to Ni─Ni/Ni─Mo coordination. Compared with Ni‐NiO@SC and Ni@SC, Ni‐NiO‐MoO_2_@SC displays a relatively weaker Ni─Ni/Ni─Mo signal at high k‐values, while the Ni─O peak remains prominent. For the Mo K‐edge (Figure 3h), the main intensity at k ≈ 6.8 Å^−1^, R ≈ 1.95 Å arises from Mo─O scattering, and the peak at k ≈ 10.2 Å^−1^, R ≈ 2.50 Å is attributed to Mo─Mo/Mo─Ni coordination. In comparison with MoO_2_@SC, the relative intensity distribution of Mo─O and Mo─Mo/Mo─Ni peaks in Ni‐NiO‐MoO_2_@SC is distinctly modulated. Meanwhile, a new scattering signal at high k‐values (k > 9 Å^−1^) can be unambiguously assigned to Ni─Mo interfacial coordination, which is absent in binary and single‐component samples. All the above results further demonstrate that the multi‐component heterointerfaces of Ni‐NiO‐MoO_2_@SC enable the modulation of the local coordination environment, which in turn optimizes the interfacial electronic structure of the multi‐phase heterojunction.

Then, X‐ray photoelectron spectroscopy (XPS) spectra were taken to elucidate the elemental compositions and electronic configurations in Ni‐NiO‐MoO_2_@SC (Figures S17 and S18). Figure 3i reveals three characteristic Ni 2p_3/2_/2p_1/2_ states in Ni‐NiO‐MoO_2_@SC: metallic Ni^0^ (852.7/870.2 eV), oxidized Ni^2+^ (856.2/873.7 eV), and corresponding satellite features (861.6/879.7 eV). Parallel analysis of Ni‐NiO‐MoO_2_@SC in Figure 3j demonstrates multivalent Mo speciation, with deconvoluted 3d_5/2_/3d_3/2_ doublets spanning Mo^0^ (228.3/231.3 eV), Mo^4+^ (229.5/232.5 eV), Mo^5+^ (232.7/235.7 eV), and Mo^6+^ (230.6/233.6 eV) states. The detected metallic Mo^0^ likely originates from reductive phase formation during annealing, whereas higher oxidation states suggest surface auto‐oxidation upon air exposure. This mixed‐valent configuration could enhance catalytic functionality via synergistic electron transfer mechanisms.

More importantly, the peaks of Ni 2p in Ni‐NiO‐MoO_2_@SC show a slight positive shift compared with those of Ni@SC and Ni‐NiO@SC, indicating that Ni has lost electrons and acted as an electron donor in the heterostructure (Figure 3i). Furthermore, the peaks of Mo 3d in Ni‐NiO‐MoO_2_@SC (Figure 3j) showed different binding energy shifts in different reference systems. The Mo 3d peaks show a negative shift relative to MoO_2_@SC, indicating that Mo gains electrons in the heterostructure. In contrast, when compared with Ni‐NiO‐MoO_2_, Mo is positively shifted, implying the introduction of SC, which further transfers electrons from the Mo site to SC. This phenomenon demonstrates the significant directional charge redistribution behavior between the interfaces: the electrons are transferred from the metal Ni to NiO and MoO_2_ sites, and eventually enriched to SC (Figure S19). This stepwise directional electron migration promotes the construction of a strong intrinsic electric field at the heterogeneous junction interface.

Ultraviolet photoelectron spectroscopy (UPS) was used to further reveal the electronic structure and energy band evolution of the catalyst interface. Combined with the UPS test results (Figure S20 and Table S5), it can be seen that Ni‐NiO‐MoO_2_@SC has a moderate work function (WF, 4.62 eV) and the lowest valence band edge position (ΔE, 1.66 eV), which confirms that the electronic interaction between the components of the heterogeneous junction coregulates the band structure and the Fermi level position, and further supports the directional charge transfer behavior and the formation of the built‐in electric field. The interfacial charge‐transport ability was optimized, providing favorable electronic‐structure conditions for the adsorption and activation of electrocatalytic reaction intermediates.

### 2.2 UOR Performance of the Catalysts

A three‐electrode system was employed to evaluate the UOR performance of Ni‐NiO‐MoO_2_@SC. Firstly, Figure S21a shows the optimal UOR activity that occurred in 1.0 M KOH with 0.5 M urea, outperforming other urea concentrations (0.1–0.9 M), thereby selecting this electrolyte for further tests. At the same current densities, Ni‐NiO‐MoO_2_@SC required lower potentials for UOR than for OER (Figure S21b). This highlights UOR's potential to replace energy‐intensive OER at the anode, significantly improving energy conversion efficiency in urea electrolysis, suggesting its promising application in sustainable hydrogen production systems.

As demonstrated in Figure 4a, Ni‐NiO‐MoO_2_@SC reaches the lowest potentials of 1.34/1.36/1.40/1.44 V at 10/100/500/1000 mA cm^−2^, respectively. Meanwhile, it exhibits the lowest Tafel slope (21.45 mV dec^−1^), which compares favorably with the values for Ni‐NiO‐MoO_2_, Ni‐NiO@SC, MoO_2_@SC, and Ni@SCIn addition, the Radar plots (Figure 4c) met the key indicators, including potential, TOF (Figure S22a), MA (Figure S22b), and ECSA (Figure S23), comprehensively evaluating the catalytic activity of UOR, all of which demonstrate the superior performance of Ni‐NiO‐MoO_2_@SC [54]. This benefits from the synergistic heterogeneous interfaces provided by Sulfur‐doped lignin‐derived carbon, Ni, NiO, and MoO_2_, which enrich the number of active sites and accelerate the reaction kinetics of the urea oxidation reaction. Notably, the as‐prepared catalyst outperforms the commercial RuO_2_/C catalyst (Figure S21c). Meanwhile, it exhibits favorable and competitive performance in both the potential at 100 mA cm^−2^ and the Tafel slope among the reported state‐of‐the‐art catalysts, demonstrating its promising potential for practical applications (Figure 4d and Table S6).

**FIGURE 4:**
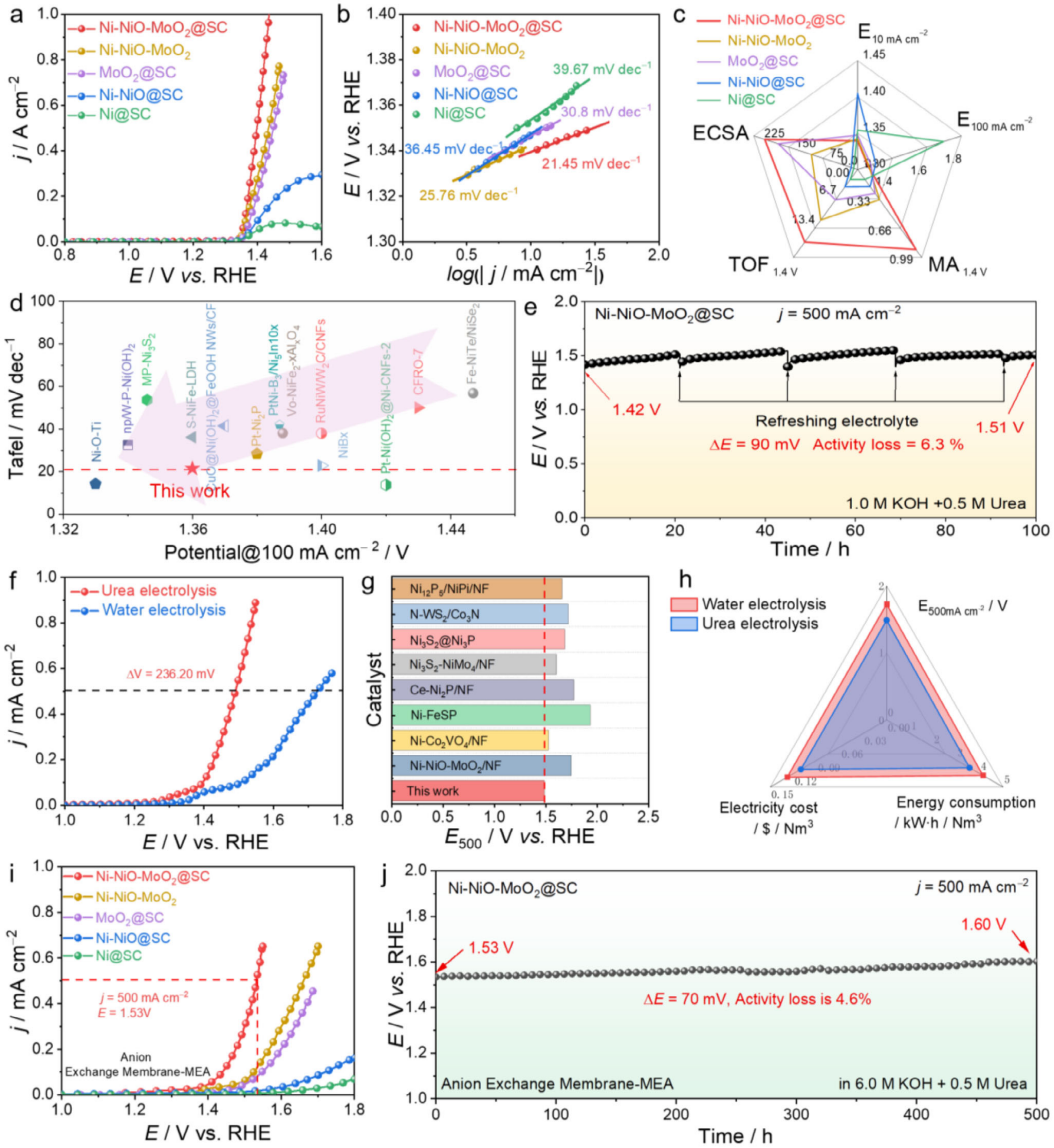
(a) Polarization curves; (b) Tafel slopes; (c) Integrated electrochemical performance radar char; (d) Comparisons of UOR potentials and Tafel [19, 35, 36, 37, 38, 39, 40, 41, 42, 43, 44, 45, 46]; (e) UOR stability; (f) Comparing urea electrolysis and water electrolysis; (g) Comparison with reported catalysts [11, 47, 48, 49, 50, 51, 52, 53]; (h) Economic indicators; (i) LSV curves of urea electrolysis of all catalysts in MEA; (j) Urea oxidation stability test of Ni‐NiO‐MoO_2_@SC in MEA.

As evidenced by chronopotentiometric measurements (Figure 4e), Ni‐NiO‐MoO_2_@SC displays exceptional stability at 500 mA cm^−2^, sustaining continuous operation for 100 h with merely 6.3% activity loss. Notably, the initial potential is 1.42 V, about 20 mV higher than the polarization‐curve value under the same conditions. This is mainly caused by a slight increase in ohmic impedance and interfacial mass‐transfer resistance due to the accumulation of transient bubbles, rather than by attenuation of the catalyst structure. In stark contrast, Ni‐NiO‐MoO_2_ without sulfur‐doped lignin‐derived carbon exhibits lower UOR activity and a rapid activity decay to 21.3% within 45 h under identical conditions (Figure S24a,b). This degradation primarily stems from structural deterioration, including fracture and collapse of both the micro‐nanorod array architecture and substrate due to severe electrochemical corrosion during prolonged electrolysis, along with partial material detachment (Figure S24c). These observations collectively demonstrate the compromised chemical stability and conclusively validate the critical protective role of sulfur‐doped lignin‐derived carbon coating in shielding active components from electrolyte corrosion.

Besides, the Ni‐NiO‐MoO_2_@SC‐powder shows poor UOR activity (Figure S25a) and 20.8% activity loss (Figure S25b), which is higher than Ni‐NiO‐MoO_2_@SC with the micro‐nano‐arrays structure. The morphological analysis (Figure S25c) attributes this deficiency to nanoparticle aggregation‐induced active site occlusion, exacerbated by binder‐induced mechanical instability during prolonged testing, suggesting that Ni‐NiO‐MoO_2_@SC has enhanced mechanical stability. Post‐stability characterization using linear sweep voltammetry (LSV), SEM, and XRD (Figure S26) confirms that the micro‐nano‐array structure in Ni‐NiO‐MoO_2_@SC preserves structural integrity, thereby enhancing mechanical stability. These findings systematically establish a strategy of sulfur‐doped lignin‐derived carbon encapsulation combined with a micro‐nano array structure to enhance the chemical/mechanical stability of the catalyst, providing a feasible approach for practical catalyst design.

Furthermore, high‐resolution XPS spectra collected before and after the UOR reaction reveal distinct changes in surface valence states (Figure S27). After the UOR stability test, the Ni 2p spectrum of Ni‐NiO‐MoO_2_@SC shows an increased proportion of Ni^3+^ assigned to in situ formed NiOOH, accompanied by a slight reduction in the relative content of pristine Ni^2+^ and Ni^0^, which correspond to NiO and Ni (Figure S27a). For the Mo 3d spectra (Figure S27b), no drastic valence state rearrangement or obvious phase transformation occurs after long‐term UOR cycling, demonstrating the excellent structural and compositional stability of the MoO_2_ component during electrocatalysis. These XPS analyses not only verify the overall structural robustness of the catalyst but also provide direct experimental evidence of the in situ formed of NiOOH active intermediates during UOR. Such results are consistent with the following in situ electrochemical characterizations and theoretical DFT calculations.

It is well known that Ni and Mo have excellent catalytic activity in the cathodic hydrogen evolution reaction, so the HER performance of Ni‐NiO‐MoO_2_@SC was assessed in a three‐electrode system. In Figures S28–S32, Ni‐NiO‐MoO_2_@SC still exhibits superior HER performance and shows promising application potential compared with other catalysts. Stability tests (Figures S33–S36) reveal that the sulfur‐doped lignin‐derived carbon coating enhances chemical stability, while the micro‐nano array structure ensures mechanical robustness, resulting in only an 8.7% performance loss and a mere 20 mV potential drop after 100 h at −500 mA cm^−2^.

Based on its bifunctional catalytic capability for UOR and HER, a two‐electrode urea electrolysis system was assembled. In Figure 4f, urea electrolysis reduces the cell voltage by 236.20 mV at 500 mA cm^−2^ compared to water electrolysis and achieves higher current densities under equal potentials, confirming UOR as an energy‐saving alternative to OER for efficient hydrogen production. Ni‐NiO‐MoO_2_@SC achieves 1.49 V at 500 mA cm^−2^, surpassing other prepared catalysts (Figure S37). Its efficient bifunctionality across wide current densities is further verified in Figure 4g and Table S8.

The gases produced during the electrolysis process were quantified using the water‐displacement method. As a cathode, Ni‐NiO‐MoO_2_@SC delivers actual hydrogen production yields nearly to theoretical values, confirming its superior HER catalytic performance (Figure S38). When functioning as the anode, the catalyst achieves a Faradaic efficiency of ∼ 92.6%, verifying that it can trigger ultra‐efficient urea oxidation with substantial suppression of parasitic side reactions (Figure S39). Excellent stability is also maintained, with over 94% performance retained after 100 h at 500 mA cm^−2^ (Figure S40). To evaluate the economic advantage of urea electrolysis‐assisted hydrogen production [55, 56], relevant economic calculations are performed under the industrial current density of 500 mA cm^−2^. The total energy consumption and electricity cost for the UOR‐assisted hydrogen production are 3.57 kW·h/Nm^3^ H_2_ and 0.1107$/Nm^3^ H_2_, respectively (Figure 4h). Compared with water electrolysis (4.14 kW·h/Nm^3^ H_2_ and 0.1283$/Nm^3^ H_2_) and the recently reported electrocatalytic systems (Table S9), this value is economically competitive and shows great potential for industrial application.

Then, a membrane electrode assembly (MEA, Figure S41) configuration was subsequently tested. In a 6.0 M KOH + 1.0 M urea electrolyte under operational conditions, Figure 4i shows that Ni‐NiO‐MoO_2_@SC achieved merely 1.53 V at a current density of 500 mA cm^−2^. These voltages reflect lower operational demands relative to Ni‐NiO‐MoO_2_, Ni‐NiO@SC, MoO_2_@SC, and Ni@SC. Remarkably, Ni‐NiO‐MoO_2_@SC maintained robust operational stability during a 500‐hour chronopotentiometric test at 500 mA cm^−2^, with only a 4.6% efficiency loss (Figure 4j), underscoring its viability for large‐scale industrial adoption.

### 2.3 Exploration of the UOR Mechanism

Operando electrochemical impedance spectroscopy (OEIS), utilizing Bode phase and Nyquist analyses, is critical for understanding UOR dynamics at electrode‐electrolyte interfaces [57]. Figure 5a demonstrates that Ni‐NiO‐MoO_2_@SC for UOR exhibits a lower Bode phase angle than OER at equivalent potentials. Strikingly, Figure 5a reveals a distinct phase angle attenuation in the low‐frequency region when UOR initiates at 1.30 V, a potential substantially lower than the 1.5 V required for OER activation. Complementary to the Nyquist data, which further confirms this trend, UOR displays smaller semicircle diameters than OER under identical conditions, indicative of faster charge‐transfer kinetics (Figure S42). This kinetic advantage aligns with Bode phase observations, collectively suggesting that UOR exhibits accelerated reaction thermodynamics compared to OER. The preferential occurrence of UOR at reduced potentials effectively suppresses competing OER pathways, thereby optimizing energy utilization. Furthermore, the Ni‐NiO‐MoO_2_@SC displays a lower Bode phase angle and reduced semicircle diameter relative to the other catalysts, implying enhanced charge‐transfer kinetics and improved interfacial reactivity (Figures S43 and S44).

**FIGURE 5:**
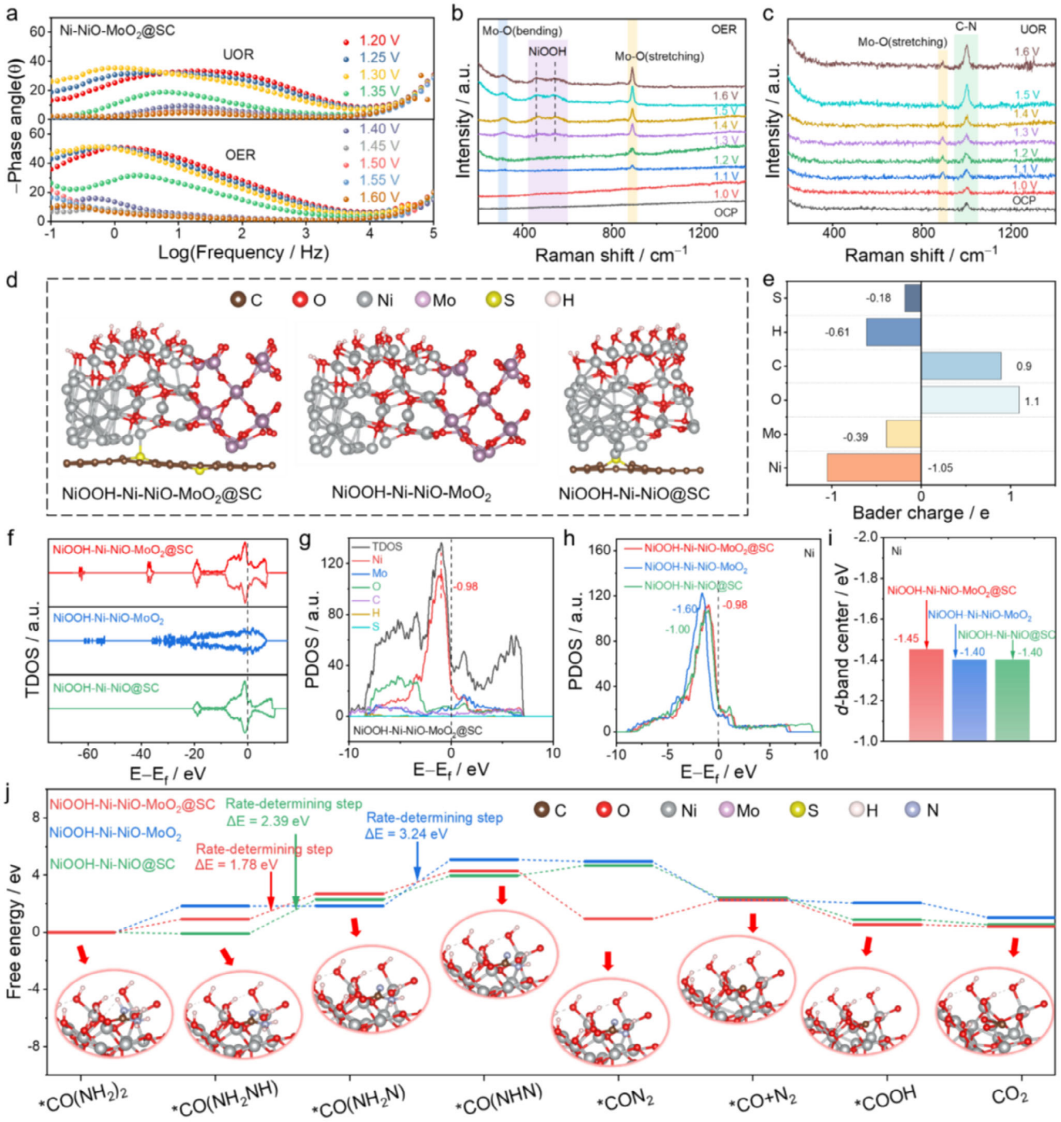
(a) In situ EIS; (b) In situ Raman in OER; (c) In situ Raman in UOR; (d) Schematic diagram of NiOOH‐Ni‐NiO‐MoO_2_@SC, NiOOH‐Ni‐NiO‐MoO_2_@SC, and NiOOH‐Ni‐NiO‐MoO_2_@SC; (e) Bader charge; (f) TDOS of prepared catalyst; (g) PDOS of NiOOH‐Ni‐NiO‐MoO_2_@SC; (h) PDOS and (i) d‐band center of the Ni 2p states for prepared catalyst; (j) Free energy of surface reconstruction on prepared catalyst in UOR reaction.

The synthesized Ni‐NiO‐MoO_2_@SC demonstrates remarkable UOR activity at a low applied potential (1.35 V), with Bode phase profiles in the mid‐to‐high frequency range implying surface‐mediated indirect oxidation mechanisms [58]. In‐situ Raman spectroscopy was employed to track the catalyst's dynamic evolution during OER and UOR (Figures 5b,c). Characteristic NiOOH peaks at 459.7 and 540.4 cm^−1^ emerged at 1.30 V during OER, confirming its role as an active intermediate. In contrast, these peaks were absent in the UOR process. To clarify this phenomenon, time‐resolved in situ Raman measurement was further performed (Figure S45). In 1.0 M KOH at 1.3 V, NiOOH peaks gradually intensified from 2 to 4 min, verifying continuous generation under anodic polarization. Upon urea addition at 6 min, NiOOH signals vanished, accompanied by the appearance of a urea C−N peak at ∼1000 cm^−1^. As urea was depleted at 10 min, NiOOH peaks reappeared. This result demonstrates that NiOOH formed in situ is rapidly consumed by urea molecules, establishing a fast dynamic equilibrium that keeps NiOOH concentration below the Raman detection limit.

Differentiated structural evolution can also be reflected from Mo−O vibration signals. The Mo─O stretching mode near 892 cm^−1^ exists in both OER and UOR, while the 310 cm^−1^ Mo─O bending vibration only appears in OER. This contrast confirms that Ni‐NiO‐MoO_2_@SC has a milder interfacial oxidation environment during UOR, which not only realizes fast consumption and regeneration of NiOOH active sites, but also effectively suppresses the excessive reconstruction of Mo‐based components. Such favorable dynamic structural behavior endows the catalyst with superior low‐potential UOR kinetics and well‐maintained structural stability during long‐term operation.

To elucidate the intrinsic UOR reaction mechanism over Ni‐NiO‐MoO_2_@SC, NiOOH sites were identified as the dominant active sites, and the adsorption behavior of urea molecules and the corresponding reaction energy barriers were investigated via DFT calculations. In‐situ EIS and in‐situ Raman results verify that the UOR process follows a surface‐mediated oxidation mechanism, in which the rate‐determining step and charge transfer are mainly governed by NiOOH species derived from activated nickel‐based surface sites. Accordingly, NiOOH‐Ni‐NiO‐MoO_2_@SC was established as a theoretical model that matches the actual catalytic phase composition (Figure 5d) [58, 59].

Then, differential charge density analysis was employed to unveil the charge transfer within Ni‐NiO‐MoO_2_@SC. Figure S46 clearly illustrates the charge transfer across the multiphase heterointerfaces in NiOOH‐Ni‐NiO‐MoO_2_@SC. Meanwhile, by analyzing the Bader charge values of the key atoms at the interface and the overall average Bader charge value (Figure 5e), it can be concluded that nickel, molybdenum, and sulfur lose electrons, while carbon and oxygen gain electrons, indicating that electron transfer occurs from Ni‐NiO‐MoO_2_ to the sulfur‐doped carbon layer. This aligns with the previously proposed interfacial charge‐transfer mechanism based on XAFS, XPS, and UPS results. Such electron redistribution makes nickel on the surface of Ni‐NiO‐MoO_2_@SC more prone to lose electrons and be oxidized to form high‐valence‐state NiOOH.

Total density of states (DOS) analysis (Figure 5f) indicates that NiOOH‐Ni‐NiO‐MoO_2_@SC exhibits the highest density near the Fermi level. This electronic structure feature is a direct theoretical signature of the multiphase heterointerface‐induced synergy. The strong electronic interaction at the interface gives rise to two primary effects [60, 61]: (1) substantial lattice strain that upshifts the *d*‐band center of the active metal sites (e.g., Ni, Figure 5i), and (2) a built‐in electric field that drives charge redistribution across the interface. Collectively, these effects lead to electron delocalization and the formation of electron‐deficient/rich active sites, resulting in the observed prominent DOS at the Fermi level (Figure 5g). The projected density of states (PDOS) of the Ni 2p orbitals (Figure 5h) reveals a reduced electron density of Ni in NiOOH‐Ni‐NiO‐MoO_2_@SC, compared with NiOOH‐Ni‐NiO‐MoO_2_ and NiOOH‐Ni‐NiO@SC, confirming the optimization of the electronic structure through the synergistic effects of the multiphase heterojunction interfaces. This optimized electronic structure significantly enhances charge transfer kinetics and optimizes six‐electron steps, including processes such as proton‐coupled electron transfer (PCET), N─N bond coupling, C─N bond cleavage, and adsorption/desorption of intermediates (*COO, *CO(NH)_2_, etc.), thereby accounting for the superior UOR activity observed experimentally.

According to the reaction mechanism of urea (Figure S47), constructed the free energy diagram was constructed (Figure 5j), and the results show that the rate‐determining step for NiOOH‐Ni‐NiO‐MoO_2_@SC is the step *CO(NH_2_NH) → *CO(NH_2_N) (ΔE = 1.78 eV), which is superior to NiOOH‐Ni‐NiO@SC (*CO(NH_2_NH) → *CO(NH_2_N), ΔE = 2.39 eV) and NiOOH‐Ni‐NiO‐MoO_2_ (*CO(NH_2_N) → *CO(NHN), ΔE = 3.24 eV). This result unambiguously confirms that the synergistic integration of MoO_2_ and sulfur‐doped carbon is crucial for facilitating the N─H bond cleavage, thereby drastically enhancing the UOR kinetics. Moreover, the step *CO(NHN) → *CON_2_ of NiOOH‐Ni‐NiO‐MoO_2_@SC exhibits a spontaneous reaction with ΔE < 0, distinct from other catalysts, which indicates that NiOOH‐Ni‐NiO‐MoO_2_@SC is more prone to N─H bond cleavage and N─N bond coupling during UOR, while NiOOH‐Ni‐NiO@SC and NiOOH‐Ni‐NiO‐MoO_2_ are more prone to C─N bond cleavage. Thus, NiOOH‐Ni‐NiO‐MoO_2_@SC demonstrates the best UOR catalytic activity. Furthermore, the poisoning effect of key intermediates on the catalyst is also a critical factor for evaluation. As shown in Figure 5j, NiOOH‐Ni‐NiO‐MoO_2_@SC has the lowest *COOH desorption energy, indicating that *COOH does not excessively accumulate on the surface after urea oxidation, thereby endowing it with excellent anti‐poisoning capability and stability.

## 3 Conclusions

In summary, a multiphase heterogeneous urea electrocatalyst (Ni‐NiO‐MoO_2_@SC.) was designed through multi‐strategy assembly to achieve efficient and stable urea electrolysis at high current densities. Physical characterization and DFT results reveal that the multiphase heterointerfaces, formed by sulfur‐doped lignin‐derived carbon encapsulating Ni, NiO, and MoO_2_, induce lattice strain and built‐in electric fields. Lattice strain primarily modulates the local lattice environment, changes bond lengths, and upshifts the d‐band center to optimize intermediate adsorption. Built‐in electric field mainly drives directional charge transfer across interfaces, promotes electron delocalization, and accelerates the six‐electron UOR process. Such dual modulation synergistically enhances electron transfer across the heterointerfaces, enables the dynamic formation of NiOOH to avoid surface passivation, and lowers the energetic barriers for urea adsorption and activation. Benefiting from the buffered interfacial oxidation regulated by MoO_2_, the system facilitates effective urea adsorption by overcoming a low energy barrier (ΔE = 1.78 eV), ensures selective cleavage of N─H bonds, and promotes efficient N─N bond coupling. Additionally, it prevents early C─N bond breakage and limits the buildup of intermediates like NiOOH and *COOH, significantly enhancing the UOR catalytic performance.

Furthermore, the sulfur‐doped lignin‐derived carbon encapsulated strategy synergistically works with the micro‐nanoarray architecture to enhance both chemical and mechanical stability. These key characteristics enable the Ni‐NiO‐MoO_2_@SC catalyst to achieve a highly efficient surface‐mediated UOR process (1.34/1.40 V@10/500 mA cm^−2^). As a bifunctional catalyst, Ni‐NiO‐MoO_2_@SC achieves 500 mA cm^−2^ at 1.53 V and maintains this performance for 500 h with minimal decay. This strategy provides a scalable blueprint for designing Ni‐based catalysts applicable to urea electrolysis and biomass conversion.

## 4 Experimental Methods

### 4.1 Synthesis of Catalysts

The Ni‐NiO‐MoO_2_@SC catalyst was synthesized via a hydrothermal process followed by subsequent calcination. Briefly, ammonium molybdate tetrahydrate (512 mg), nickel nitrate hexahydrate (840 mg), and sodium lignin sulfonate (400 mg) were dissolved in 30 mL of deionized water with a piece of Ni foam (NF) in a 50 mL autoclave. After hydrothermal treatment at 180°C for 6 h and cooling, the sample was washed, dried, and then calcined at 500°C for 2 h under an H_2_/Ar (5:95 v/v) atmosphere.

Control samples were prepared by modifying this procedure: Ni‐NiO‐MoO_2_ (without sodium lignin sulfonate); MoO_2_@SC (without nickel nitrate hexahydrate); Ni‐NiO@SC (without ammonium molybdate, calcined at 400°C); Ni@SC (without ammonium molybdate, calcined at 600°C); Control samples were prepared by correspondingly modifying the above synthetic procedure while keeping all other reaction parameters identical: Ni‐NiO‐MoO_2_ (synthesized in the absence of sodium lignin sulfonate); MoO_2_@SC (without nickel nitrate hexahydrate); Ni‐NiO@SC (without ammonium molybdate, calcined at 400°C); Ni@SC (without ammonium molybdate, calcined at 600°C). For the Ni‐NiO‐MoO_2_@SC‐powder sample, the precursor was synthesized identically without the addition of NF during the hydrothermal reaction to collect pure powder products after calcination. Prior to electrochemical testing, the resultant powder catalyst was dispersed in a mixed solution of ethanol, deionized water, and 5 wt.% Nafion solution, ultrasonicated to form a homogeneous ink, and then uniformly coated onto the NF surface for electrode fabrication.

### 4.2 Physicochemical Characterization

The structural and chemical properties of the catalytic materials were systematically analyzed using a suite of advanced characterization techniques: X‐ray diffraction (XRD, Rigaku) with Cu_Kα_ radiation (λ = 0.15406 nm), scanning electron microscopy (SEM, Hitachi SU8220), high‐resolution transmission electron microscopy (HRTEM, FEI Tecnai G2 F20) with lattice resolution, inductively coupled plasma mass spectrometry (ICP‐MS, PerkinElmer NexION 300X), energy‐dispersive X‐ray spectroscopy (EDS, Ametek), X‐ray photoelectron spectroscopy (XPS, Thermo Scientific K‐Alpha), ultraviolet photoelectron spectroscopy (UPS, Thermo ESCALAB QXi, He I radiation, 21.22 eV), Raman spectroscopy (Horiba Jobin Yvon) with 532 nm He/Ne laser excitation. X‐ray absorption spectroscopy data were collected at the BL14W1 station in Shanghai Synchrotron Radiation Facility (SSRF).

### 4.3 Synchrotron Radiation Data Analysis Methods

Synchrotron radiation X‐ray absorption fine structure (XAFS) data were collected at the Ni K–edge and Mo K–edge at the BL14W1 beamline of the Shanghai Synchrotron Radiation Facility (SSRF). The data were recorded in fluorescence mode for Ni–NiO–MoO_2_@SC and reference samples. The acquired data were processed using the ATHENA and ARTEMIS modules in the IFEFFIT software package [62]. The pre–edge background was subtracted, and the post–edge background was normalized using standard procedures. The χ(k) functions were Fourier transformed into R‐space with a k–weight of 3 to obtain radial distribution functions. Wavelet transform (WT) analysis was performed using the HAMA program to distinguish the backscattering atoms and radial distances. All fitting parameters were constrained within reasonable ranges to ensure reliability.

### 4.4 Electrochemical Measurements

Electrochemical assessments, including UOR, HER, and urea electrolysis, were conducted on a DH7001B electrochemical workstation employing three‐electrode configurations or two‐electrode assemblies. Testing utilized either baseline 1.0 M KOH electrolyte or urea‐enriched alkaline media (1.0 M KOH + 0.5 M urea), with a standardized electrode arrangement: working electrode of catalyst, graphite counter electrode, and Hg/HgO reference electrode. All measured potentials underwent conversion to the reversible hydrogen electrode (RHE) scale through Nernstian correction*E*(*vs*.RHE) = *E*(*vs*.Hg/HgO) + 0.059*pH + 0.098 [26]. Correction voltage was subjected to *iR*_s_ compensation by the specific formula (*E*_corrected_ = *E*_uncorrected_ − *iR_s_* *0.9), which accounts for corrected voltage (*E*_corrected_), measured voltage (*E*_uncorrected_), solution resistance (*R*_s_), and current (*i*). For the 100‐hour three‐electrode long‐term stability test, intermittent electrolyte replenishment was performed throughout the measurement to counteract water evaporation and urea consumption in the non‐sealed H‐type cell, ensuring constant electrolyte composition and testing conditions. For the 500‐hour MEA durability test implemented in a closed circulating pumping setup, the same electrolyte (1.0 M KOH + 0.5 M urea) was periodically supplemented to compensate for reactant loss caused by the UOR process. Continuous electrolyte circulation effectively homogenizes concentration distribution near electrodes, and no obvious potential disturbance appears on chronopotentiometry curves upon supplementation.

### 4.5 DFT Calculation

DFT calculations were performed using the Vienna Ab initio Simulation Package (VASP) within the framework of the projector augmented‐wave (PAW) method. The exchange‐correlation interaction was described by the generalized gradient approximation (GGA) using the Perdew‐Burke‐Ernzerhof (PBE) functional. The theoretical model, denoted as NiOOH‐Ni‐NiO‐MoO_2_@SC, was constructed on a sulfur‐doped graphitic carbon support (SC), in which one carbon atom in the graphitic carbon layer was substituted by one sulfur atom. The support exposed the graphite C (001) basal plane. Two‐layer Ni (100), NiO (100), and MoO_2_ (100) slabs were sequentially assembled through a lattice‐matching strategy to construct the Ni‐NiO‐MoO_2_ heterostructure. Appropriate surface supercells were selected to ensure that the lattice mismatch between adjacent components was below 5%. Subsequently, a single‐layer NiOOH overlayer exposing the NiOOH (001) basal plane was placed on the Ni/NiO interfacial region, forming the final NiOOH‐Ni‐NiO‐MoO_2_@SC heterostructure. A vacuum region of approximately 32 Å was introduced along the surface‐normal (c) direction to eliminate interactions between periodically repeated slabs. During structural optimization, the bottom two atomic layers of the slab were fixed to simulate the bulk constraint, while all remaining atoms were fully relaxed until the total energy converged to 1.0 × 10^−5^ eV and the residual force on each atom was less than 0.02 eV Å^−1^. The Brillouin zone was sampled using a Γ‐centered 3×1×1 Monkhorst‐Pack k‐point mesh.

The reaction free energy change (Δ*G*) was evaluated under thermodynamic equilibrium according to the following relationship:

$$\Delta G=\Delta E_{DFT}+\Delta E_{ZPE}-T\Delta S$$

where Δ*E*_DFT_is the electronic energy difference obtained from DFT calculations, Δ*E*_ZPE_ is the zero‐point energy correction estimated within the quantum harmonic oscillator approximation, and Δ*S*denotes the entropy contribution derived from vibrational frequency analysis at 298.15 K. All optimized geometries correspond to local minima on the potential energy surface with minimized total energy.

### 4.6 Economic Analysis

Based on electricity costs, the energy consumption of water electrolysis and urea electrolysis is calculated to estimate the production cost of H_2_. We performed exemplary calculations for the operating current density of 500 mA cm^−2^ to determine the hydrogen production costs. The electricity consumption per Nm^3^ of H_2_ produced was calculated as follows [55]:

$$W=\frac{E_{total}}{V_{H_{2}}}=\frac{\left(U*i*t\right)*10^{-3}}{\left(n_{H_{2}}*Vm\right)*10^{-3}}=\frac{U*i*t}{\frac{Q}{u*F}*Vm}$$

where E_total_ is the total power consumed, t = 1 h when it is in E_total_, n is the molarity of electrons transferred for the product, U is the input voltage, i is the operating current density, Q is the total amount of charge that passes through, and Vm is the molar volume of the gas at standard atmospheric pressure (22.4 mol L^−1^). F is Faraday's constant. It is noted that n = 2 for urea oxidation coupled with the hydrogen evolution reaction (For every mole of urea consumed, 3 moles of hydrogen gas are produced).

## Author Contributions

The manuscript was written with contributions from all authors.

## Conflicts of Interest

The authors declare no competing financial interests or personal relationships with the editorial board that could have appeared to influence the work reported in this paper.

## Supporting Information

The Supporting information contains of the Crystalline composition and structure calculation, the Economic analysis, SEM, BET, Contact angles, Optical imaging of gaseous release behavior, Raman, XRD, XPS, UPS, LSV, TOF, MA, ECSA, Stability test, in‐situ EIS plots, and charge density image.

## Supporting information




**Supporting File 1**: adma74790‐sup‐0001‐SuppMat.docx.

**Supporting File 2**: adma74790‐sup‐0002‐SuppMat.mov.

**Supporting File 3**: adma74790‐sup‐0003‐SuppMat.mov.

## Data Availability

All data required to evaluate the conclusions of this study are presented within the article and its Supplementary Information. Additional datasets related to this work are available from the corresponding author upon reasonable request. The source data for all figures and analyses are included in the manuscript.
