# Supplementary material for: Lattice Strain and Built‐In Field Synergistically Boost Urea Oxidation via Dynamic NiOOH Mediation at Multiphase Heterointerfaces
Source: Adv Mater. 2026 Sep 1;38(60):e74790. doi: 10.1002/adma.74790 (PMC13712197; doi:10.1002/adma.74790)
Supplement: Supplementary file 1 [file ADMA-38-e74790-s001.docx]

Supplementary Material

**Lattice Strain and Built-In Field Synergistically Boost Urea Oxidation via Dynamic NiOOH Mediation at Multiphase Heterointerfaces**

Jiawei Li ^1#^, Liancen Li ^1#^, Guangfu Qian ^1*^, Zishan Cai ^1^, Yiping Cao ^1^, Yihao Xu ^1^,

Ruyu Zhang ^1^, Jinli Chen ^2*^, Changzhou Chen ^1*^, Panagiotis Tsiakaras ^3*^

^1^ Guangxi Key Laboratory of Clean Pulp & Papermaking and Pollution Control, School of Light Industry and Food Engineering, Guangxi University, Nanning 530004, China.

^2^ State Key Laboratory of Materials Processing & Die and Mould Technology, School of Materials Science and Engineering, Huazhong University of Science and Technology, Wuhan 430074, China.

^3^ Laboratory of Alternative Energy Conversion Systems, Department of Mechanical Engineering, School of Engineering, University of Thessaly, Pedion Areos 38834, Greece.

***Corresponding authors: qianguangfu@gxu.edu.cn (G. Qian), chenjinli@hust.edu.cn (J. Chen), chenchangzhou@gxu.edu.cn (C. Chen), tsiak@uth.gr (P. Tsiakaras).

^#^ These authors contributed to the work equally and should be regarded as co-first authors.

1. **Results and Discussion**

**1.1 Crystalline composition and structure calculation**

*Compositional and structural analyses of the Ni-NiO-MoO_2_@SC* (**Tables S1-S3**): The XRD spectra were analyzed by referencing standard PDF cards for Ni (PDF #04-0850), NiO (PDF #47-1049), and MoO_2_ (PDF #32-0671). For the MoO_2_ (−111) plane, the 2θ peaks of Ni-NiO-MoO_2_@SC and MoO_2_@SC were observed at 26.13° and 26.03°, corresponding to interplanar spacings of 0.3410 nm (0.41% compressive strain) and 0.3423 nm (0.03% tensile strain) from the standard reference. Similarly, the NiO (111) plane exhibited 2θ shifts at 36.95° (Ni-NiO-MoO_2_@SC) and 37.64° (Ni-NiO@SC), yielding lattice spacings of 0.2433 nm (0.87% tensile strain) and 0.2390 nm (0.91% compressive strain). Further analysis of the Ni (200) plane showed peaks at 51.49° (Ni-NiO-MoO_2_@SC), 52.00° (Ni-NiO@SC), and 52.28° (Ni@SC), with corresponding spacings of 0.1775 nm (0.73% tensile strain), 0.1759 nm (0.17% compressive strain), and 0.1750 nm (0.68% compressive strain). These systematic peak shifts confirm the formation of a multi-component heterojunction and the resulting lattice strain effects, where atomic incorporation leads to compressive strain with larger atoms and tensile strain with smaller atoms, consistent with experimental observations.

**1.2 Economic analysis**

Based on electricity costs, the energy consumption of water electrolysis and urea electrolysis is calculated to estimate the production cost of H_2_. We performed exemplary calculations for the operating current density of 500 mA cm^−2^ to determine the hydrogen production costs. The electricity consumption per Nm^3^ of H_2_ produced was calculated[1] as follows:

$$\text{W}=\frac{\text{E}_{\text{total}}}{\text{V}_{\text{H}_{2}}}=\frac{\left(\text{U}\times\text{i}\times\text{t}\right)\times10^{-3}}{\left(\text{n}_{\text{H}_{2}}\times\text{V}_{\text{m}}\right)\times10^{-3}}=\frac{\text{U}\times\text{i}\times\text{t}}{\frac{\text{Q}}{\text{n}\times F}\times\text{V}_{\text{m}}}$$

Where E_total_ is the total power consumed, t = 1 hour when it is in E_total_, n is the molarity of electrons transferred for the product, U is the input voltage, i is the operating current density, Q is the total amount of charge that passes through, and V_m_ is the molar volume of the gas at standard atmospheric pressure (22.4 mol L^−1^). F is Faraday’s constant. It is noted that n =2 for urea oxidation coupled with the hydrogen evolution reaction (For every mole of urea consumed, 3 moles of hydrogen gas are produced).

For UOR: $\text{W}=\frac{2\times96485\times1.49}{3600\times22.4}=3.57$ (kW·h / Nm^3^)

For OER: $\text{W}=\frac{2\times96485\times1.73}{3600\times22.4}=4.14$ (kW·h / Nm^3^)

The electricity cost is assumed to be 0.031 $/kWh [2]. So, the Electricity cost (H_2_ per kg):

For UOR=3.57×0.031=0.1107 ($ / Nm^3^)

For OER=4.14×0.031=0.1283 ($ / Nm^3^)

**Table S9** summarizes cell voltage and specific energy consumption of Ni-NiO-MoO_2_@SC versus recently reported urea electrolysis electrocatalysts. Varied cost calculation protocols across literature preclude unified cost comparison; thus, energy consumption serves as the sole comparative metric. Lower cell voltage and energy consumption relative to most benchmark catalysts confirm the superior energy efficiency and economic potential of the material.

2. **Supplementary Figures**

**Figure S1.** (a-b) SEM images of NF.

**Figure S2.** (a-b) SEM images of the Precursor.

**Figure S3.** (a-b) SEM images of Ni-NiO-MoO_2_.

**Figure S4.** (a-b) SEM images of MoO_2_@SC.

**Figure S5.** (a-b) SEM images of Ni-NiO@SC.

**Figure S6.** (a-b) SEM images of Ni@SC.

**Figure S7.** (a-b) SEM images of Ni-NiO-MoO_2_@SC-powder.

**Figure S8.** (a-c) The N_2_ adsorption/desorption curves; (d-f) Pore size profiles of prepared catalysts.

**Figure S9.** (a-c) The N_2_ adsorption/desorption curves;(d-f) Pore size profiles of prepared catalysts.

、

**Figure S10.** The BET surface of prepared catalysts.

**Figure S11.** (a-e) Contact angles of all samples in 1.0 M KOH + 0.5 M urea solution.

**Figure S12**. (a-e) Gaseous H_2_ release behavior of all samples at 0, 5, and 10 s at 50 mA cm^−2^ in 1.0 M KOH + 0.5 M urea solution.

**Figure S13**. (a-e) Gaseous H_2_ release behavior of all samples at 0, 5, and 10 s at 500 mA cm^−2^ in 1.0 M KOH + 0.5 M urea solution.

**Figure S14.** Raman image of prepared catalysts.

**Figure S15.** (a-e) XRD images of prepared catalysts.

**Figure S16.** (a-b) The TEM image of another area of Ni-NiO-MoO_2_@SC.

**Figure S17.** (a) XPS spectra for the prepared catalysts; (b) Local magnification of S 2p on the XPS spectrum.

**Figure S18.** The XPS spectrum of C 1s, O 1s, and S 2p on Ni-NiO-MoO_2_@SC.

The C 1s signals of Ni-NiO-MoO_2_@SC/NF (**Figure S18a**) were resolved at 284.8 (C−C), 285.6 (C−S), and 289.0 (C=O), while O 1s spectra (**Figure S18b**) exhibited peaks at 530.4 (metal−O), 531.3 (lattice−O), and 532.8 (adsorbed−O). **Figure S18c** shows that the peaks of S 1s can be vested in C−SO_x_−C (166.7/169.1/170.1 eV), S 2p_3/2_/2p_1/2_ (163.2/164.7 eV), and metal-S (162.3 eV).

**Figure S19.** The interfacial charge-transfer mechanism of Ni-NiO-MoO_2_@SC.

**Figure S20.** (a-e) UPS spectra and (f) Comprehensive energy-band diagram.

To reveal the electronic structure and interfacial charge characteristics of the catalysts, ultraviolet photoelectron spectroscopy (UPS) was performed to determine the work function (WF) and valence band edge position (ΔE) of a series of samples (**Figure S20**). WF was calculated from the secondary electron cutoff edge, while the ΔE was obtained by extrapolating the tangent line of the valence band rise. All parameters are summarized in **Table S5**.

For SC, the WF is 4.77 eV, and the ΔE is 3.42 eV. After introducing metallic Ni to form Ni@SC, the WF decreases significantly to 1.92 eV, and the ΔE increases to 3.50 eV. This change arises because metallic Ni, as a strong electron donor, injects a large number of free electrons into the sulfur-doped carbon, shifting the Fermi level toward the conduction band and lowering the surface electron escape barrier. When MoO_2_ is introduced to form MoO_2_@SC, the WF rises to 3.03 eV, with ΔE decreasing to 2.88 eV. This is attributed to the high density of free electrons and high intrinsic conductivity of MoO_2_, which contribute additional charge carriers to the SC system, resulting in an electron concentration between that of SC and Ni@SC. For Ni-NiO@SC, the WF increases significantly to 5.36 eV, while the ΔE decreases to 2.60 eV. This is due to semiconductor NiO acting as an electron acceptor, which traps and consumes free electrons at the interface, causing the Fermi level to shift toward the valence band and increasing the difficulty of surface electron escape.

The Ni-NiO-MoO_2_@SC exhibits a WF of 4.62 eV and a significantly reduced ΔE of 1.66 eV, indicating more complex charge redistribution at the ternary heterojunction interface. Specifically, electrons are directionally transferred from metallic Ni to NiO and MoO_2_, and finally migrate to SC, forming a strong built-in electric field at the interface and coordinately modulating the Fermi level to a suitable position close to the valence band edge. This electronic structure not only optimizes the surface electron escape ability but also facilitates the transition of valence band electrons and the activation of reaction intermediates during electrocatalysis, providing direct evidence at the electronic structure level for the excellent catalytic performance of the main sample.

**Figure S21.** (a-b) UOR LSV curves of Ni-NiO-MoO_2_@SC at different urea concentrations; the UOR LSV curves of Ni-NiO-MoO_2_@SC compare with (c) RuO_2_/C and (d) bare nickel foam.

**Figure S21a** shows the optimal UOR activity that occurred in 1.0 M KOH with 0.5 M urea, outperforming other urea concentrations (0.1-0.9 M), thereby selecting this electrolyte for further tests. Notably, the LSV revealed negligible current density in pure 0.5 M urea solution (**Figure S21b**), indicating that KOH’s superior conductivity is essential for efficient current generation. The results underscore the critical role of alkaline conditions in facilitating UOR kinetics.

**Figure S22.** (a-b) TOF and MA of Ni-NiO-MoO_2_@SC in UOR.

**Figure S23.** (a) The CV curve of Ni-NiO-MoO_2_@SC; (b) the relationship between the corresponding capacitance current and the scanning rate; (c) the LSV curves normalized by ECSA.

**Figure S24.** (a) UOR LSV curves of Ni-NiO-MoO_2_@SC and Ni-NiO-MoO_2_; (b) UOR stability of Ni-NiO-MoO_2_/NF; (c) SEM images of Ni-NiO-MoO_2_ after UOR stability test.

**Figure S25.** (a) UOR LSV curves of Ni-NiO-MoO_2_@SC and Ni-NiO-MoO_2_@SC-powder; (b) UOR stability of Ni-NiO-MoO_2_@SC-powder; (c) SEM images of Ni-NiO-MoO_2_@SC-powder after the UOR stability test.

As shown in Figure S25c, the blurry appearance of the micrographs arises from severe surface charging effects during SEM measurement. After prolonged alkaline UOR electrolysis, partial degradation of the insulating Nafion binder aggravates surface charge accumulation under electron beam irradiation, resulting in focal drift and distorted imaging. All SEM testing parameters were unified for all samples to ensure comparative consistency.

**Figure S26. (a)** LSV curves of Ni-NiO-MoO_2_@SC before and after the UOR CP stability test; (b) Comparison of XRD before and after the UOR stability test of Ni-NiO-MoO_2_@SC; (c) SEM images of Ni-NiO-MoO2@SC after the UOR stability test.

**Figure S27.** The XPS spectrum competition of before and after the UOR stability test of Ni-NiO-MoO_2_@SC in (a) Ni 2p and (b) Mo 3d.

**Figure S28**. HER LSV curves at (a) different urea concentrations of Ni-NiO-MoO_2_@SC and (b) the prepared catalyst; (c) Tafel slopes; (d) HER LSV curves of Ni-NiO-MoO_2_@SC and Pt/C.

**Figure S29**. Comparisons of HER potentials and Tafel [3-15].

Similarly, to explore the HER activity under different solutions, **Figure S28a** shows that the addition of urea has no significant effect on the catalyst’s HER performance. Subsequently, the HER kinetic behavior of the Ni-NiO-MoO_2_@SC composite was systematically investigated in an alkaline urea medium (1.0 M KOH + 0.5 M urea). As revealed in **Figures S28b-S28c**, Ni-NiO-MoO_2_@SC reaches low potentials of −20.98 mV@−10 mA cm^−2^, −64.48 mV@−100 mA cm^−2^, and −154.27 mV@−500 mA cm^−2^ with a Tafel slope of 66.93 mV dec⁻^1^, surpassing the respective values of the other prepared samples, including Ni-NiO-MoO_2_, Ni-NiO@SC, MoO_2_@SC, and Ni@SC. This performance not only competes with the commercial Pt/C benchmark but surpasses most transition metal-based HER catalysts (**Figure S28d, Figure S29, Table S7**), highlighting efficient kinetics and industrial potential.

**Figure S30**. (a-b) TOF and MA of Ni-NiO-MoO_2_@SC in HER.

Moreover, **Figure S30** exhibits the TOF and MA. At −200 mV, Ni-NiO-MoO_2_@SC achieves a TOF of 25.54 s^−1^, which is 1.27×, 19×, 2.68× and 53× higher than Ni-NiO-MoO_2_ (20.01 s^−1^), Ni-NiO@SC (1.31 s^−1^), MoO_2_@SC (9.52 s^−1^), and Ni@SC (0.49 s^−1^), respectively. Concurrently, Ni-NiO-MoO_2_@SC’s MA at −200 mV reaches 1408.34 mA mg⁻^1^, corresponding to 3.36-57.93 × enhancements over Ni-NiO-MoO_2_ (419.49 mA mg^−1^), Ni-NiO@SC (377.53 mA mg^−1^), MoO_2_@SC (377.53 mA mg^−1^), and Ni@SC (24.31 mA mg^−1^), respectively.

**Figure S31.** (a-d) Bode plots of prepared catalysts at different voltages.

**Figure S32.** (a-d) Nyquist plots of prepared catalysts at different voltages.

Particularly, in-situ EIS is employed to discern the elementary reactions during the HER process. Given that electron transfer takes place exclusively in the Volmer and Heyrovsky stages, the Tafel step, lacking electron transfer, cannot be detected by in-situ EIS. Ni-NiO-MoO_2_@SC presents two phase angles, indicating a Volmer-Heyrovsky HER pathway (**Figure S31a**), accompanied by a Tafel slope of 66.93 mV dec^−1^. Although this pathway is demonstrated, the phase angles of Ni-NiO-MoO_2_@SC diminish as the potential rises, in contrast to Ni-NiO-MoO_2_, Ni-NiO@SC, MoO_2_@SC, and Ni@SC (**Figure S31**), suggesting better interface charge transfer among Ni, NiO, MoO_2_, and sulfur-doped lignin-derived carbon in Ni-NiO-MoO_2_@SC. Moreover, the Nyquist plots of Ni-NiO-MoO_2_@SC (**Figure S32**) at identical potentials are consistent with the Bode phase plots of other catalysts.

These findings collectively confirm four critical advantages: (1) Multiphase heterogeneous interfaces among the sulfur-doped lignin-derived carbon, Ni, NiO, and MoO_2_ optimize hydrogen adsorption energetics through interfacial electron redistribution and lattice distortion, effectively lowering the Heyrovsky step energy barrier; (2) Micro-nano-arrays and multiphase heterogeneous interfaces collectively enhance intrinsic activity and increases the number of active sites; (3) Hierarchical porosity prevents nanoparticle aggregation, maintaining sustained interfacial reactivity; (4) Multiphase heterogeneous interfaces among the sulfur-doped lignin-derived carbon, Ni, NiO, and MoO_2_ accelerate the interfacial reactions and electron transfer during the HER process.

**Figure S33**. HER stability of Ni-NiO-MoO_2_@SC.

**Figure S34.** (a) LSV curves of Ni-NiO-MoO_2_@SC before and after the HER CP stability test; (b) Comparison of XRD before and after the HER stability test of Ni-NiO-MoO_2_@SC; (c) SEM images of Ni-NiO-MoO_2_@SC after the HER stability test.

**Figure S35.** (a) HER LSV curves of Ni-NiO-MoO_2_@SC and Ni-NiO-MoO_2_@SC-powder; (b) HER stability of Ni-NiO-MoO_2_@SC-powder; (c) SEM images of Ni-NiO-MoO_2_@SC-powder after the HER stability test.

**Figure S36.** (a) HER LSV curves of Ni-NiO-MoO_2_@SC and Ni-NiO-MoO_2_; (b) HER stability of Ni-NiO-MoO_2_; (c) SEM images of Ni-NiO-MoO_2_ after the HER stability test.

To verify the mechanical and chemical stability of Ni-NiO-MoO_2_@SC, tests were carried out on Ni-NiO-MoO_2_@SC, powder Ni-NiO-MoO_2_@SC, and Ni-NiO-MoO_2_ without sulfur-doped lignin-derived carbon at −500 mA cm^−2^ in 1.0 M KOH and 0.5 M urea solutions. The Ni-NiO-MoO_2_@SC electrode demonstrated significantly enhanced electrochemical activity for HER, as evidenced by linear sweep voltammetry (LSV) measurements, outperforming both the powder-Ni-NiO-MoO_2_@SC and Ni-NiO-MoO_2_ counterparts (**Figures S35a and S36a**). **Figure S33** shows that Ni-NiO-MoO_2_@SC can operate stably for 100 hours with a potential change of 20 mV, outperforming powder Ni-NiO-MoO_2_@SC (**Figure S35b**) and Ni-NiO-MoO_2_ (**Figure S36b**). Post-test morphological analysis (**Figure S34**) confirmed that the rod-shaped microarrays and nanoparticles of Ni-NiO-MoO_2_@SC largely retained their original architecture. In contrast, the powdered Ni-NiO-MoO_2_@SC and Ni-NiO-MoO_2_ suffered severe corrosion, structural fracture, and nanoparticle aggregation (**Figures S35c and S36c**), accompanied by dramatic activity losses of 70.7% and 66.8%, respectively, within 50 hours. These results unequivocally demonstrate that the synergistic integration of sulfur-doped lignin-derived carbon with micro-nano arrays enhances the catalyst’s electrochemical and mechanical stability under harsh conditions.

**Figure S37.** Urea electrolysis LSV for the prepared catalysts.

**Figure S38.** The volume of hydrogen produced by Ni-NiO-MoO_2_@SC and the amount of H_2_ theoretically calculated and experimentally measured versus time at −50 mA cm^−2^.

**Figure S39.** The volume of N_2_+CO_2_ produced by Ni-NiO-MoO_2_@SC and the amount of N_2_+CO_2_ theoretically calculated and experimentally measured versus time at 500 mA cm^−2^.

**Figure S40.** Stability test of urea electrolysis at 10 mA cm^−2^ and 500 mA cm^−2^ in Ni-NiO-MoO_2_@SC.

**Figure S41.** Schematic diagram of MEA device.

**Figure S42.** (a-b) Nyquist plots of Ni-NiO-MoO_2_@SC at different voltages.

**Figure S43.** (a-d) Bode plots of prepared catalysts at different voltages.

**Figure S44.** (a-d) Nyquist plots of prepared catalysts at different voltages.

**Figure S45.** Time-resolved in-situ Raman spectroscopy of Ni-NiO-MoO_2_@SC.

**Figure S46.** Charge density and the Bader charge values of the key atoms at the interface of NiOOH-Ni-NiO-MoO_2_@SC.

**Figure S47.** Schematic diagram of the urea reaction equation.

2. **Supplementary Tables**

**Table S1.** Diffraction information of Ni-NiO-MoO_2_@SC and MoO_2_@SC at the MoO_2_ (−111) crystal surface.

| **MoO_2_ (**−**111)** | **Peak /°** | **d /nm** | **d_standard_ /nm** | **Strain degree** |
| --- | --- | --- | --- | --- |
| Ni-NiO-MoO_2_@SC | 26.13 | 0.3410 | 0.3420 | 0.41% (compress) |
| MoO_2_@SC | 26.03 | 0.3423 | 0.3420 | 0.03% (tensile) |

**Table S2.** Diffraction information of Ni-NiO-MoO_2_@SC and Ni-NiO@SC at the NiO (111) crystal surface.

| **NiO (111)** | **Peak /°** | **d /nm** | **d_standard_ /nm** | **Strain degree** |
| --- | --- | --- | --- | --- |
| Ni-NiO-MoO_2_@SC | 36.95 | 0.2433 | 0.2412 | 0.87% (tensile) |
| Ni-NiO@SC | 37.64 | 0.2390 | 0.2412 | 0.91% (compress) |

**Table S3.** Diffraction information of Ni-NiO-MoO_2_@SC, Ni-NiO@SC and Ni@SC at the Ni (200) crystal surface.

| **Ni (200)** | **Peak /°** | **d /nm** | **d_standard_ /nm** | **Strain degree** |
| --- | --- | --- | --- | --- |
| Ni-NiO-MoO_2_@SC | 51.49 | 0.1775 | 0.1762 | 0.73% (tensile) |
| Ni-NiO@SC | 52.00 | 0.1759 | 0.1762 | 0.17% (compress) |
| Ni@SC | 52.28 | 0.1750 | 0.1762 | 0.68% (compress) |

**Table S4.** Elemental composition of the samples obtained from ICP-MS.

| **Elements**  **Catalysts** | **S** **(wt.%)** | **Ni** **(wt.%)** | **Mo** **(wt.%)** |
| --- | --- | --- | --- |
| Ni-NiO-MoO_2_@SC | 0.79 | 6.41 | 4.85 |
| Ni-NiO-MoO_2_ | N/A | 8.53 | 10.37 |
| MoO_2_@SC | 2.62 | N/A | 10.77 |
| Ni-NiO@SC | 1.83 | 9.97 | N/A |
| Ni@SC | 1.4 | 10.23 | N/A |

**Table S5.** The UPS test results of the samples.

| **Catalysts** | **WF** **(****eV)** | **E_Cotoff_** **(****eV)** | **ΔE** **(eV)** |
| --- | --- | --- | --- |
| Ni-NiO-MoO_2_@SC | 4.62 | 16.60 | 1.66 |
| MoO_2_@SC | 3.03 | 18.19 | 2.88 |
| Ni-NiO@SC | 5.36 | 15.86 | 2.6 |
| Ni@SC | 1.92 | 19.30 | 3.5 |
| SC | 4.62 | 16.45 | 3.42 |

**Table S6.** Summary of recently reported UOR potentials corresponding to catalysts.

| Catalysts | Potential @ *j* (V) | References |
| --- | --- | --- |
| Ni-NiO-MoO_2_@SC | 1.34@10  1.36@100  1.40@500  1.44@1000 | This work |
| Ni-O-Ti | 1.30@10  1.33@100 | [16] |
| Mn-Ni_2_P | 1.435@1000 | [17] |
| S-NiFe-LDH | 1.36@100 | [18] |
| np/W-P-Ni(OH)_2_ | 1.34@100 | [19] |
| N-Co_9_S_8_/Ni_3_S_2_/NF | 1.44@300  1.47@400 | [20] |
| Ni_3_N-VN | 1.33@100 | [21] |
| P-W-NiCo | 1.30@300 | [8] |
| TAPP(Fe)-OFPc(Ni)-COF | 1.37@10 | [22] |
| Pt-Ni_2_P | 1.38@100 | [23] |
| Ag-CoOOH@NiOOH | 1.33@100 | [24] |
| Ni_2_Fe(CN)_6_ | 1.35@100 | [25] |
| Ni_3_N/FeNi_3_N/NF | 1.4@300 | [26] |
| WN/Ni_3_C | 1.33@50 | [27] |
| 2D CoSe_2_/Ni_0.85_Se | 1.33@100 | [28] |
| Ni_3_Se_2_@NiMoO_4_/NF | 1.31@100 | [29] |
| Ni_2_P_4_O_12_/NiTe | 1.313@10 | [14] |
| MoS_2_ NPs/CoS_2_ NTs | 1.31@10  1.44@50 | [30] |
| Mo/MoS_V1_ | 1.49@100 | [31] |
| Ni_2_P–Co_2_P/C | 1.32@100 | [32] |
| NiCoVO_x_ | 1.40@100 | [33] |
| Ni_2_P/NiF_2_/PCNF | 1.39@10 | [34] |
| La-O-Ni | 1.49@1000 | [35] |
| Ni-Fe-P@Ni-Se | 1.30@10 | [36] |

**Table S7.** Summary of recently reported HER potentials corresponding to catalysts.

| Catalysts | Potential @ *j* (mV) | References |
| --- | --- | --- |
| Ni-NiO-MoO_2_@SC | −20.98@−10  −64.99@−100  −154.62@−500  −230.16@−1000 | This work |
| Mo1-NT@NF | −272@−100  −384@−200 | [37] |
| NF/NiMoO_4_-S | −73@−10 | [38] |
| P-W-NiCo | −85@−10  −211@−100 | [8] |
| N_i3_S_2_@Ni_3_P | −332@−500 | [9] |
| N-Ni-MoO_2_/NF | −49@−10  −242@−500  −284@−1000 | [7] |
| Ni/W_5_N_4_/NF | −205@−500 | [39] |
| Ni_2_P_4_O_12_/NiTe | −24@−10 | [14] |
| NiMoO_4_/NF | −64@−10 | [40] |
| CNT/MoC/NMC-3 | −51@−10 | [41] |
| Pt-Ni(OH)_2_@Ni-CNFs-2 | −20.8@−10  −107.8@−200 | [42] |
| NiSe_2_/MoSe_2_ | −70@−10 | [43] |
| Ni/ZnO | −34@−10 | [44] |
| W-NT@NF-3 | −88@−10 | [45] |
| NiIr(OH)_2_-EO | −26@−10 −69@−100 | [46] |
| Mn-Ni_2_P | −190.5@−100 | [17] |
| Ni_3_N/FeNi_3_N/NF | −48@−10  −102@−100 | [26] |
| NiCo_2_S_4_/CoMo_2_S_4_ | −120@−10  −270@−100 | [47] |
| CoNi-LSCF | −28@−10 | [48] |
| C@NiCoP/NF | −168@−100 | [49] |
| Ni/MoBTx | −32@−10 | [50] |
| NiMoO_4_ | −238@−10 | [51] |

**Table S8.** Summary of recently reported urea-assist water electrolysis potentials corresponding to catalysts.

| Catalysts | Potential @ *j* (V) | References |
| --- | --- | --- |
| Ni-NiO-MoO_2_@SC | 1.39@100  1.49@500 | This work |
| N-Ni-MoO_2_/NF | 1.571@100  1.753@500 | [7] |
| NiIr(OH)_2_-EO | −26@−10 −69@−100 | [46] |
| P-W-NiCo | 1.36@10  1.45@300 | [8] |
| Pt-Ni(OH)_2_@Ni-CNFs-2 | 1.400@10  1.544@50 | [42] |
| N-Co_9_S_8_/Ni_3_S_2_/NF | 1.79@100 | [20] |
| C@NiCoP/NF | 1.51@100 | [49] |
| NiB_x_ | 1.66@100 | [52] |
| Ni_2_P/Ni_3_N/NCNF | 1.39@10 | [53] |
| Ni_3_S_2_@Ni_3_P | 1.69@500 | [9] |
| CNT/MoC/NMC-3 | 1.291@10 | [41] |
| MoS_2_ NPs/CoS_2_ NTs | 1.65@10  1.85@50 | [30] |
| Ni-NC@DWC | 1.13 @100 | [54] |
| P/Cr_60_-NiMoO_4_ | 1.35@10  1.46@50  1.50@100 | [55] |
| Ni_2_P_4_O_12_/NiTe | 1.475@100 | [14] |
| Ni_2_P/NiF_2_/PCNF | 1.39@10 | [34] |
| CoS_1.097_/Ni_3_S_2_ | 1.57@100 | [3] |
| Ni_3_Se_2_@NiMoO_4_/NF | 1.35@10 | [29] |
| Vo-NiMoOOH/N | 2.71@1000 | [1] |
| F-NiO/Ni@C | 1.37@10 | [56] |

**Table S9.** Comparison of cell voltage and specific energy consumption for urea electrolysis between Ni-NiO-MoO_2_@SC and recently reported representative electrocatalysts.

| Catalysts | Potential @ *j* (V) | W / (kW·h/Nm^3^) | References |
| --- | --- | --- | --- |
| Ni-NiO-MoO_2_@SC | 1.49@500 | 3.57 | This work |
| Vo-NiMoOOH/NF | 2.1@500 | 5.02 | [1] |
| Cr-Ni_3_S_2_ | 1.55@500 | 3.7 | [57] |
| Ni^Ⅱ+δ^-O-Co^Ⅱ+δ^ | 1.58@100 | 3.68 | [58] |
| S-NiFe-LDH | 1.47@100 | 3.52 | [18] |
| Co_3_CuN/Fe_x_N_y_ | 1.8@500 | 4.32 | [59] |
| Ni(OH)_x_-HCO_3_ | 1.61@400 | 3.6 | [60] |

Note: Energy consumption of Co_3_CuN/Fe_x_N_y_ is originally provided as 48.11 kWh per gasoline-gallon equivalent hydrogen (kWh/GGE H_2_). To facilitate intuitive and unified comparison among different electrocatalytic systems, the unit is converted to kWh per standard cubic meter of produced hydrogen (kWh/Nm^3^). The lower heating value of hydrogen under standard conditions is 10.79 MJ Nm^−3^, and the total calorific value corresponding to 1 GGE equals 120.2 MJ[61]. Accordingly, 1 GGE of hydrogen is equivalent to approximately 11.14 standard cubic meters of hydrogen.

**5. References**

[1] T. Chen, J. X. Song, M. L. Xu, Z. F. Li, *Oxygen vacancies activate electrophilic-nucleophilic dual sites in Mo-doped NiOOH for hydrogen evolution via urea-assisted electrocatalysis approaching industrial-level current densities*, *Appl. Catal. B Environ.* **2026**, 382, 126012, DOI: 10.1016/j.apcatb.2025.126012.

[2] J. Lu, Z. Yu, X. Wei, X. Zhang, X. Wang, K. Liu, Y. Cai, H. Pan, D. Liu, Z. Wang, *Fe/Co co‐doping engineering for corrosion‐resistant and effective seawater electrolysis*, *Adv. Mater.* **2025**, 38, e15156, DOI: 10.1002/adma.202515156.

[3] M. X. Du, Y. J. Ji, Y. Y. Li, S. Z. Liu, J. Q. Yan, *Construction of an internal charge field: CoS_1.097_/Ni_3_S_2_ heterojunction promotes efficient urea oxidation reaction*, *Adv. Funct. Mater.* **2024**, 34, 2402776, DOI: 10.1002/adfm.202402776.

[4] L. N. Sha, T. F. Liu, K. Ye, K. Zhu, J. Yan, J. L. Yin, G. L. Wang, D. X. Cao, *A heterogeneous interface on NiS@Ni_3_S_2_/NiMoO_4_ heterostructures for efficient urea electrolysis*, *J. Mater. Chem. A* **2020**, 8, 18055, DOI: 10.1039/d0ta04944a.

[5] K. Xiong, L. J. Yu, Y. Xiang, H. D. Zhang, J. Chen, Y. Gao, *Cerium-incorporated Ni_2_P nanosheets for enhancing hydrogen production from overall water splitting and urea electrolysis*, *J. Alloys Compd.* **2022**, 912, 165234, DOI: 10.1016/j.jallcom.2022.165234.

[6] X. D. Ding, L. S. Pei, Y. X. Huang, D. Y. Chen, Z. L. Xie, *Hollow NiCoP nanoprisms derived from prussian blue analogues as bifunctional electrocatalysts for urea-assisted hydrogen production in alkaline media*, *Small.* **2022**, 18, 2205547, DOI: 10.1002/smll.202205547.

[7] G. F. Qian, T. Lu, Y. P. Wang, H. T. Xu, X. Y. Cao, Z. H. Xie, C. Z. Chen, D. Y. Min, *N-induced compressive strain in Ni-MoO_2_ heterostructure with micro-nano array for improving high-current-output urea-assisted water electrolysis performance*, *Chem. Eng. J.* **2024**, 480, 147993, DOI: 10.1016/j.cej.2023.147993.

[8] Q. Y. Chen, X. Deng, H. P. He, L. Jiang, C. Liang, J. Zhang, R. L. Wang, J. W. Chen, *Modulating nanostructure and electronic structure of P and W co-doped carbon encapsulated heterovalent NiCo composite for accelerating urea oxidation reaction*, *Chem. Eng. J.* **2024**, 484, 149561, DOI: 10.1016/j.cej.2024.149561.

[9] X. Guo, L. Y. Qiu, M. G. Li, F. Y. Tian, X. Ren, S. Jie, S. Geng, G. H. Han, Y. R. Huang, Y. Song, W. W. Yang, Y. S. Yu, *Accelerating the generation of NiOOH by in-situ surface phosphating nickel sulfide for promoting the proton-coupled electron transfer kinetics of urea electrolysis*, *Chem. Eng. J.* **2024**, 483, 149264, DOI: 10.1016/j.cej.2024.149264.

[10] D. F. Wen, M. Ye, Y. Xia, W. S. Mai, Z. Q. Zhang, W. T. Zhang, W. D. Peng, W. Hu, L. H. Tian, *CoP nanoplates dotted with porous Ni_3_S_2_ nanospheres for the collaborative enhancement of hydrogen production via urea-water electrolysis*, *Appl. Surf. Sci.* **2022**, 586, 152736, DOI: 10.1016/j.apsusc.2022.152736.

[11] J. Wu, X. Yang, J. Zhang, S. Guan, J. Han, J. Wang, K. Li, G. Zhang, T. Guan, *Surface engineering of Ni_2_P/CoP nanosheet heterojunctions by the formation of F-doped carbon layers for boosting urea-rich water electrolysis*, *J. Power Sources* **2022**, 548, DOI: 10.1016/j.jpowsour.2022.232065.

[12] X. Y. Zhuo, W. J. Jiang, T. Q. Yu, G. F. Qian, J. L. Chen, H. F. Yang, S. B. Yin, *Crystalline-amorphous Ni_3_S_2_-NiMoO_4_ heterostructure for durable urea electrolysis-assisted hydrogen production at high current density*, *ACS Appl. Mater. Interfaces* **2022**, 14, 46481, DOI: 10.1021/acsami.2c11238.

[13] X. J. Xu, P. Y. Du, T. Guo, B. L. Zhao, H. L. Wang, M. H. Huang, *In situ grown Ni phosphate@Ni_12_P_5_ nanorod arrays as a unique core-shell architecture: Competitive bifunctional electrocatalysts for urea electrolysis at large current densities*, *ACS Sustainable Chem. Eng.* **2020**, 8, 7463, DOI: 10.1021/acssuschemeng.0c01814.

[14] P. Guo, S. F. Cao, W. J. Huang, X. Q. Lu, W. Z. Chen, Y. Z. Zhang, Y. J. Wang, X. Xin, R. Q. Zou, S. B. Liu, X. H. Li, *Heterojunction-induced rapid transformation of Ni^3+^/Ni^2+^ sites which mediates urea oxidation for energy-efficient hydrogen production*, *Adv. Mater.* **2024**, 36, 2311766, DOI: 10.1002/adma.202311766.

[15] B. Wang, L. Guo, J. Zhang, Y. Qiao, M. He, Q. Jiang, Y. Zhao, X. Shi, F. Zhang, *Synthesis of nickel nitride‐based 1D/0D heterostructure via a morphology‐inherited nitridation strategy for efficient electrocatalytic hydrogen evolution*, *Small.* **2022**, 18, 2201927, DOI: 10.1002/smll.202201927.

[16] G. M. Zhan, L. F. Hu, H. Li, J. Dai, L. Zhao, Q. Zheng, X. Y. Zou, Y. B. Shi, J. X. Wang, W. Hou, Y. C. Yao, L. Z. Zhang, *Highly selective urea electrooxidation coupled with efficient hydrogen evolution*, *Nat. Commun.* **2024**, 15, 5918, DOI: 10.1038/s41467-024-50343-8.

[17] Z. H. Zhao, Y. R. Dong, H. R. Ding, X. Li, X. H. Chang, *Manganese-facilitated dynamic active-site generation on Ni_2_P with self-termination of surface reconstruction for urea oxidation at high current density*, *Water Res.* **2024**, 253, 121266, DOI: 10.1016/j.watres.2024.121266.

[18] T. T. Li, Z. Y. Zheng, Z. R. Chen, M. T. Zhang, Z. X. Liu, H. Chen, X. Xiao, S. G. Wang, H. T. Qu, Q. J. Fu, L. Liu, M. Zhou, B. R. Wang, G. M. Zhou, *Ultrafast synthesis of an efficient urea oxidation electrocatalyst for urea-assisted fast-charging Zn–air batteries and water splitting*, *Energy Environ. Sci.* **2025**, 18, 4996, DOI: 10.1039/d5ee01064k.

[19] L. B. Cai, H. Y. Bai, J. L. Li, F. Xie, K. Jiang, Y. R. Lu, H. Pan, Y. W. Tan, *Single-atom tungsten doping induced chemical–electrochemical coupled pathway on Ni(OH)_2_ enables efficient urea electrooxidation*, *Energy Environ. Sci.* **2025**, 18, 2415, DOI: 10.1039/d4ee05340k.

[20] H. Xie, Y. F. Feng, X. Y. He, Y. Zhu, Z. Y. Li, H. H. Liu, S. Y. Zeng, Q. Z. Qian, G. Q. Zhang, *Construction of nitrogen-doped biphasic transition-metal sulfide nanosheet electrode for energy-efficient hydrogen production via urea electrolysis*, *Small.* **2023**, 19, 2207425, DOI: 10.1002/smll.202207425.

[21] C. W. Ji, H. M. Duan, C. H. Wang, G. Z. Liang, X. J. Long, X. L. She, R. R. Zhang, F. L. Gong, D. H. Li, D. J. Yang, J. Liu, *Pairing N-vacancy and adjacent Ni-sites in the local microenvironment to regulate the urea oxidation reaction pathway with enhanced kinetics*, *Adv. Mater.* **2025**, 37, e2503879, DOI: 10.1002/adma.202503879.

[22] G. Y. Qiao, B. L. Wang, Z. Y. Zhao, H. D. Yu, J. Y. Lin, Y. Y. Guo, J. H. Wang, L. Li, T. Heine, D. H. Mei, E. Q. Jin, *Three-dimensional porphyrin and phthalocyanine-based covalent organic frameworks for boosting urea oxidation*, *Angew. Chem. Int. Ed.* **2025**, 64, e202508783, DOI: 10.1002/anie.202508783.

[23] J. C. Zhang, J. R. Feng, J. X. Zhu, L. Q. Kang, L. S. Liu, F. Guo, J. Li, K. Q. Li, J. Chen, W. Zong, M. Q. Liu, R. W. Chen, I. P. Parkin, L. J. Mai, G. Q. He, *Regulating reconstruction-engineered active sites for accelerated electrocatalytic conversion of urea*, *Angew. Chem. Int. Ed.* **2024**, 63, e202407038, DOI: 10.1002/anie.202407038.

[24] M. J. Wu, Y. H. Xu, J. Luo, S. Y. Yang, G. X. Zhang, L. Du, H. X. Luo, X. Cui, Y. K. Yang, S. H. Sun, *A rechargeable urea-assisted Zn-air battery with high energy efficiency and fast-charging enabled by engineering high-energy interfacial structures*, *Angew. Chem. Int. Ed.* **2024**, 63, e202410845, DOI: 10.1002/anie.202410845.

[25] S. K. Geng, Y. Zheng, S. Q. Li, H. Su, X. Zhao, J. Hu, H. B. Shu, M. Jaroniec, P. Chen, Q. H. Liu, S. Z. Qiao, *Nickel ferrocyanide as a high-performance urea oxidation electrocatalyst*, *Nat. Energy* **2021**, 6, 904, DOI: 10.1038/s41560-021-00899-2.

[26] Y. C. Dong, Y. T. Wu, X. Y. Wang, H. Wang, J. W. Ren, P. Wang, L. Pan, G. Q. Wang, R. F. Wang, *Biomimicry-inspired fish scale-like Ni_3_N/FeNi_3_N/NF superhydrophilic/superaerophobic nanoarrays displaying high electrocatalytic performance*, *Nanoscale.* **2023**, 15, 1813, DOI: 10.1039/d2nr05911h.

[27] Y. H. Feng, N. A. Ran, X. L. Wang, Q. N. Liu, J. C. Wang, L. J. Liu, K. Suenaga, W. W. Zhong, R. G. Ma, J. J. Liu, *Nanoparticulate WN/Ni_3_C coupling in ceramic coatings for boosted urea electro-oxidation*, *Adv. Energy Mater.* **2023**, 2302452, DOI: 10.1002/aenm.202302452.

[28] F. O. Boakye, M. G. Sendeku, A. Kumar, S. Ajmal, K. A. Owusu, K. B. Ibrahim, M. Tabish, F. U. Zaman, M. A. Mushtaq, K. M. Alotaibi, M. Z. Ansari, G. Yasin, *Engineering active sites on binary metal selenide heterointerface catalyst to boost urea electrooxidation*, *Appl. Catal. B Environ.* **2024**, 352, 124013, DOI: 10.1016/j.apcatb.2024.124013.

[29] Y. B. Yue, Y. Wang, N. Jian, S. Liu, Z. Wang, H. Z. Li, H. Zhao, W. F. Cai, *Built‐in electric field engineering at heterojunction interfaces for high‐performance urea electrocatalysis*, *Adv. Funct. Mater.* **2025**, e17125, DOI: 10.1002/adfm.202517125.

[30] T. L. Luyen Doan, D. C. Nguyen, K. Kang, A. Ponnusamy, H. I. Eya, N. Y. Dzade, C. S. Kim, C. H. Park, *Advanced Mott-Schottky heterojunction of semi-conductive MoS_2_ nanoparticles/metallic CoS_2_ nanotubes as an efficient multifunctional catalyst for urea-water electrolysis*, *Appl. Catal. B Environ.* **2024**, 342, 123295, DOI: 10.1016/j.apcatb.2023.123295.

[31] F. L. Gong, Z. L. Chen, C. Q. Chang, M. Song, Y. Zhao, H. T. Li, L. H. Gong, Y. L. Zhang, J. Zhang, Y. H. Zhang, S. Z. Wei, J. Liu, *Hollow Mo/MoS(Vn) nanoreactors with tunable built-in electric fields for sustainable hydrogen production*, *Adv. Mater.* **2025**, 37, e2415269, DOI: 10.1002/adma.202415269.

[32] Y. Liao, Y. Y. Chen, L. Li, S. Luo, Y. Qing, C. H. Tian, H. Xu, J. X. Zhang, Y. Q. Wu, *Ultrafine homologous Ni_2_P-Co_2_P heterostructures via space-confined topological transformation for superior urea electrolysis*, *Adv. Funct. Mater.* **2023**, 33, 2303300, DOI: 10.1002/adfm.202303300.

[33] J. Y. Zhang, P. T. Wang, M. Zheng, R. K. Hocking, Y. Zheng, S. Z. Qiao, *Dynamic regeneration of catalytic sites on V-modified NiCo oxide for boosting urea electrooxidation*, *ACS Catal.* **2025**, 15, 3143, DOI: 10.1021/acscatal.4c07778.

[34] C. Yin, J. X. Li, S. L. Wang, H. Wen, F. L. Yang, L. G. Feng, *Carbon fiber confined mixed Ni‐based crystal phases with interfacial charge redistribution induced by high bond polarity for electrochemical urea‐assisted hydrogen generation*, *Carbon Energy.* **2024**, 6, e553, DOI: 10.1002/cey2.553.

[35] X. Y. Qiang, Y. X. Yao, J. Yin, P. F. Da, Z. R. Mu, K. E. Shen, Y. M. Sun, Y. X. Zhang, P. Q. Li, Z. L. Li, P. X. Xi, C. H. Yan, *Activating La-O-Ni bridge in ordered macroporous interface for electrochemical urea wastewater purification*, *Angew. Chem. Int. Ed.* **2025**, e202424014, DOI: 10.1002/anie.202424014.

[36] K. Muzammil, F. M. A. Altalbawy, D. Sur, S. Ballal, J. Tham, A. Vijay Kumar, S. Bobonazarovna Formanova, I. Samir Alalaq, F. H. Alsultany, S. Hassan Zain Al-Abdeen, M. Alhedrawe, *Controlled designing of NiFeP@Ni-Se arrays as efficient and robust bifunctional electrocatalyst for hydrogen production coupled via urea oxidation*, *Fuel.* **2025**, 394, 135166, DOI: 10.1016/j.fuel.2025.135166.

[37] M. Y. Liu, W. H. Zou, J. Cong, N. Su, S. L. Qiu, L. X. Hou, *Identifying and unveiling the role of multivalent metal states for bidirectional UOR and HER over Ni, Mo-trithiocyanuric based coordination polymer*, *Small.* **2023**, 2302698, DOI: 10.1002/smll.202302698.

[38] D. F. Wen, W. D. Peng, W. T. Zhang, Y. Xia, M. Ye, W. Hu, *Hetero-structured NiMoO_4_/Ni_3_S_4_/MoS_2_ pompons decorated nickel foam electrode for high-efficient urea and urine electrolysis*, *Appl. Surf. Sci.* **2023**, 608, 155166, DOI: 10.1016/j.apsusc.2022.155166.

[39] Y. M. Zhou, B. X. Chu, Z. J. Sun, L. H. Dong, F. Wang, B. Li, M. G. Fan, Z. J. Chen, *Surface reconstruction and charge distribution enabling Ni/W_5_N_4_ Mott-Schottky heterojunction bifunctional electrocatalyst for efficient urea-assisted water electrolysis at a large current density*, *Appl. Catal. B Environ.* **2023**, 323, 122168, DOI: 10.1016/j.apcatb.2022.122168.

[40] B. Liu, H. M. Wen, B. Da, X. Y. Liang, K. Li, Z. H. Xu, X. D. Zhang, Y. T. Zhang, Y. C. Wang, H. Li, K. Yan, *Effective electrocatalytic xylose oxidation coupling hydrogen production on hierarchical microcolumn NiMoO_4_ array*, *Appl. Catal. B Environ.* **2025**, 375, 125443, DOI: 10.1016/j.apcatb.2025.125443.

[41] X. S. Liu, K. A. Deng, P. Liu, X. B. Lv, W. Tian, K. Ma, H. J. Li, J. Y. Ji, *Mutual promotion by structural design and intrinsic activity coupling of CNTs/MoC/CoNiMo for water splitting and urea electrolysis*, *Appl. Catal. B Environ.* **2024**, 343, 123470, DOI: 10.1016/j.apcatb.2023.123470.

[42] M. X. Zhong, M. J. Xu, S. Y. Ren, W. M. Li, C. Wang, M. B. Gao, X. F. Lu, *Modulating the electronic structure of Ni(OH)_2_ by coupling with low-content Pt for boosting the urea oxidation reaction enables significantly promoted energy-saving hydrogen production*, *Energy Environ. Sci.* **2024**, 17, 1984, DOI: 10.1039/d3ee03398h.

[43] X. C. Xu, H. J. Liao, L. Huang, S. J. Chen, R. Wang, S. T. Wu, Y. X. Wu, Z. P. Sun, H. T. Huang, *Surface reconstruction and directed electron transport in NiSe_2_/MoSe_2_ Mott-Schottky heterojunction catalysts promote urea-assisted water splitting*, *Appl. Catal. B Environ.* **2024**, 341, 123312, DOI: 10.1016/j.apcatb.2023.123312.

[44] L. L. Wang, X. R. Wang, H. J. Wang, C. Zhang, J. J. Li, G. J. Feng, X. X. Cheng, X. R. Qin, Z. Y. Yu, T. B. Lu, *Tailoring lewis acidity of metal oxides on nickel to boost electrocatalytic hydrogen evolution in neutral electrolyte*, *J. Am. Chem. Soc.* **2025**, 147, 7555, DOI: 10.1021/jacs.4c16596.

[45] M. Y. Liu, W. H. Zou, S. L. Qiu, N. Su, J. Cong, L. X. Hou, *Active site tailoring of Ni-based coordination polymers for high-efficiency dual-functional HER and UOR catalysis*, *Adv. Funct. Mater.* **2024**, 34, 2310155, DOI: 10.1002/adfm.202310155.

[46] Z. Lin, Q. L. Gao, C. Lin, X. P. Liu, Q. F. Zhang, P. Diao, *Activating and stabilizing hydrogen evolution on trace-iridium-incorporated nickel hydroxide for energy-efficient hydrogen production*, *Appl. Catal. B Environ.* **2025**, 378, 125641, DOI: 10.1016/j.apcatb.2025.125641.

[47] N. Nwaji, B. Fikadu, M. Osial, M. Warczak, Z. Moazzami Goudarzi, M. Gniadek, S. Asgaran, J. Lee, M. Giersig, *Advanced functional NiCo_2_S_4_@CoMo_2_S_4_ heterojunction couple as electrode for hydrogen production via energy-saving urea oxidation*, *Small.* **2025**, 21, e2410848, DOI: 10.1002/smll.202410848.

[48] S. Jeong, Y. H. Zhang, J. Kang, S. J. Lee, J. M. Baik, H. Park, *Pseudo-perovskite oxide with in situ surface reconstructed metals for alkaline hydrogen evolution*, *ACS Energy Letters* **2025**, 10, 3591, DOI: 10.1021/acsenergylett.5c01403.

[49] M. J. Shi, F. Sultana, X. J. Qin, P. Zhang, K. C. Qian, T. Wei, Y. Duan, T. T. Li, J. M. Bai, R. H. Li, *In situ evolution of MOF-derived C@NiCoP/NF promotes urea-assisted electrocatalytic hydrogen production*, *Appl. Catal. B Environ.* **2025**, 371, 125210, DOI: 10.1016/j.apcatb.2025.125210.

[50] H. L. Su, W. Liao, S. X. Mo, H. J. Wang, Y. H. Cao, H. Yu, H. F. Wang, *Decoupling volmer and tafel steps via dual‐site interface engineering in Ni/MoBTx for efficient hydrogen electrocatalysis*, *Adv. Funct. Mater.* **2025**, e18373, DOI: 10.1002/adfm.202518373.

[51] B. Chang, X. Y. Liu, S. W. Zuo, Y. F. Ren, J. T. He, D. Q. Wang, Y. J. Lei, M. Hu, W. L. Li, M. A. Khan, R. Aleisa, R. M. Hu, Y. Hou, H. Liu, W. J. Zhou, Z. P. Lai, H. N. Alshareef, H. B. Zhang, *Dynamic construction of a durable epitaxial catalytic layer for industrial alkaline water splitting*, *Nat. Commun.* **2025**, 16, 7959, DOI: 10.1038/s41467-025-63361-x.

[52] Y. T. Hu, L. Shao, Z. Q. Jiang, L. Shi, Q. J. Li, K. Q. Shu, H. Chen, G. D. Li, Y. Dong, T. Z. Wang, J. H. Li, L. F. Jiao, Y. D. Deng, *Unveiling the role of boron on nickel‐based catalyst for efficient urea oxidation assisted hydrogen production*, *Adv. Funct. Mater.* **2024**, 34, 2411011, DOI: 10.1002/adfm.202411011.

[53] J. X. Li, C. Yin, S. L. Wang, B. G. Zhang, L. G. Feng, *Built-in electrophilic/nucleophilic domain of nitrogen-doped carbon nanofiber-confined Ni_2_P/Ni_3_N nanoparticles for efficient urea-containing water-splitting reactions*, *Chem. Sci.* **2024**, 15, 13659, DOI: 10.1039/d4sc01862a.

[54] Z. K. Shi, Y. Zhang, W. Guo, Z. H. Niu, Y. Chen, J. L. Huang, *Self‐encapsulated Ni nanoparticles on delignified wood carbon for efficient urea‐assisted hydrogen production*, *Adv. Funct. Mater.* **2024**, 35, 2414935, DOI: 10.1002/adfm.202414935.

[55] M. Liu, H. Zhao, X. Q. Du, X. S. Zhang, *The synthesis of Cr and P codoped NiMoO_4_ nanospheres as efficient catalyst for urea oxidation and oxygen evolution reaction*, *Int. J. Hydrogen Energy* **2023**, 48, 38143, DOI: 10.1016/j.ijhydene.2023.06.181.

[56] X. F. Xu, H. Ullah, M. Humayun, L. F. Li, X. Zhang, M. Bououdina, D. P. Debecker, K. F. Huo, D. Wang, C. D. Wang, *Fluorinated Ni‐O‐C heterogeneous catalyst for efficient urea‐assisted hydrogen production*, *Adv. Funct. Mater.* **2023**, 33, 2303986, DOI: 10.1002/adfm.202303986.

[57] C. Fan, M. Zhang, Y. Li, Y. Zhang, Y.-Q. Wang, F. Gong, J. Liu, *Lewis acid-triggered hydroxyl spillover enables selective urea electrooxidation to nitrite with concurrent energy-saving hydrogen production*, *Nat. Commun.* **2026**, 17, 1585, DOI: 10.1038/s41467-026-68302-w.

[58] C. Xie, C. Zhou, Y. Zhang, B. Zhou, Y. Yao, B. Li, J. Li, J. Bai, M. Long, K. Jiang, H. Zhu, L. Zhang, *Electron delocalization in Ni–Co active pairs for efficient and robust urea electrooxidation*, *Angew. Chem. Int. Ed.* **2026**, 65, 25119, DOI: 10.1002/anie.202525119.

[59] N. Sinha, C. Das, C. C. Er, R. R. Urkude, S. Pal, B. Ghosh, S. P. Chai, P. Roy, *Dynamic and resilient cobalt copper nitride and iron nitride heterostructure aided energy economic urea electrolysis in anion exchange membrane electrolyzer*, *Small.* **2026**, 22, e00012, DOI: 10.1002/smll.202600012.

[60] X. Liu, C. Shi, G. Zhang, R. Gao, L. Pan, X. Zhang, Z.-F. Huang, J. Gong, J.-J. Zou, *Oxyanions stabilized low-coordinated nickel species for efficient and durable nucleophilic electrooxidation*, *Nat. Commun.* **2025**, 16, 11224, DOI: 10.1038/s41467-025-66298-3.

[61] E. National Academies of Sciences, and Medicine., *The Hydrogen Economy: Opportunities Costs Barriers and R&D Needs*, The National Academies Press, Washington, DC **2004**.
